# High‐Content CRISPR Screening: Methods and Applications

**DOI:** 10.1002/mco2.70964

**Published:** 2026-09-08

**Authors:** Yike Zhang, Yimeng Zhang, Xudong Tang, Xinyu Deng, Yitian Zhou, Yanfeng Wu, Jianhua Luo

**Affiliations:** ^1^ Naval Medical University Shanghai China; ^2^ National Key Laboratory of Immunity & Inflammation Institute of Immunology Navy Medical University Shanghai China

**Keywords:** CRISPR screening, delivery systems, functional genomics, high‐content screening, immunotherapy

## Abstract

Clustered regularly interspaced short palindromic repeats (CRISPR)–Cas9 screening has become a central technology in functional genomics, enabling genome‐scale interrogation via pooled perturbations. Early CRISPR screens employed survival or simple phenotypic readouts to identify essential genes and drug resistance mechanisms. However, as biological questions have shifted toward understanding regulatory networks, cellular heterogeneity, and context‐dependent gene functions, there has been increasing demand for screening strategies capable of capturing complex cellular phenotypes beyond cell fitness. Recent advances in single‐cell sequencing, high‐content imaging, and spatial transcriptomics have expanded the resolution of CRISPR screening by enabling multidimensional phenotypic characterization following genetic perturbation. By integrating pooled perturbations with diverse readouts, these approaches systematically map targeted gene edits to transcriptional states, cellular phenotypes, and microenvironmental contexts. Meanwhile, innovations in library design, delivery, and computational pipelines have further improved the robustness and interpretability of high‐content screening platforms. This review synthesizes the methodological evolution of CRISPR screening, emphasizing advances in perturbation strategies, delivery systems, and multimodal readouts. Representative applications spanning oncology, immunotherapy, developmental biology, neurobiology, and infectious diseases are delineated to demonstrate refined gene network annotations. Additionally, existing technical bottlenecks, such as scalability, cost constraints, and in vivo limitations, are critically assessed. Finally, future directions are proposed to facilitate the development of precise medicine.

## Graphical abstract

High‐content CRISPR screening represents a paradigm shift in functional genomics, moving beyond traditional survival‐based readouts to enable multidimensional mapping of genotype–phenotype relationships. This review systematically outlines the methodological evolution of this approach, detailing advances in perturbation modalities, delivery systems, and sophisticated readout platforms. In this review, we comprehensively explore its applications across diverse fields, including the dissection of tumorigenesis, metastasis, and drug resistance in cancer biology; the optimization of immunotherapies such as CAR‐T and CAR‐NK cells; and fundamental discoveries in developmental biology, neurobiology, and infectious diseases. Furthermore, we evaluate the translational trajectory of screen‐identified targets toward clinical investigation. Finally, we discuss prevailing challenges in scalability, cost, and in vivo application, providing a perspective on future integration with deeper spatiotemporal phenotyping to accelerate precision medicine. This synthesis offers a framework for understanding the current landscape and future potential of high‐content CRISPR screening.

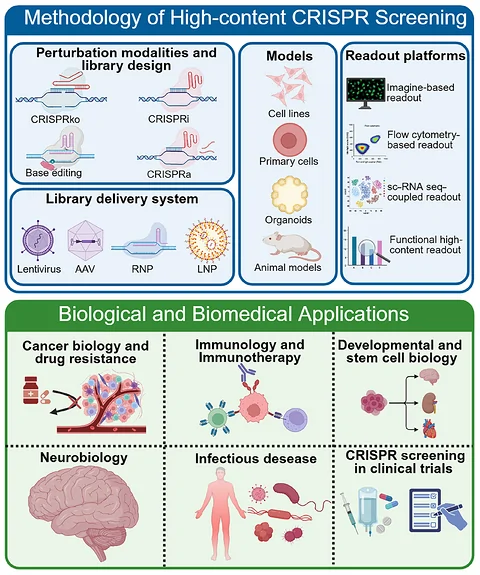

## 1 Introduction

CRISPR screening has emerged as a powerful, unbiased functional genomics tool, enabling the systematic identification of genes driving specific phenotypes or cellular pathways through genome‐scale CRISPR–Cas9 libraries. Over the past decade, this technology has been widely adopted to identify key therapeutic and functional targets, elucidating potential mechanisms across diverse fields including cell biology [1], cancer research [2], immunology [3], infectious diseases [4], microbiology [5], and neurobiology [6]. As a mature and cost‑effective tool, CRISPR screening has greatly expanded our understanding of gene function.

However, conventional CRISPR screening primarily relies on unidimensional readouts such as cell survival, proliferation, or simple fluorescent reporters, an approach that has fundamental limitations. For example, while such screens have successfully identified essential host genes for H5N1 viral replication [7], cholesterol biosynthesis targets governing stemness and drug resistance in colorectal cancer stem cells [8], and genes required for NF‐κB activation [9], they often fall short in dissecting complex biological systems, such as the tumor microenvironment (TME), neural circuits, immune cell interactions, and developmental processes. By focusing predominantly on basic survival or the expression of a limited set of genes, these screens are unable to capture intricate intracellular processes, cellular heterogeneity, and dynamic intercellular crosstalk. Addressing these limitations has become a major methodological challenge in functional genomics.

**FIGURE 1:**
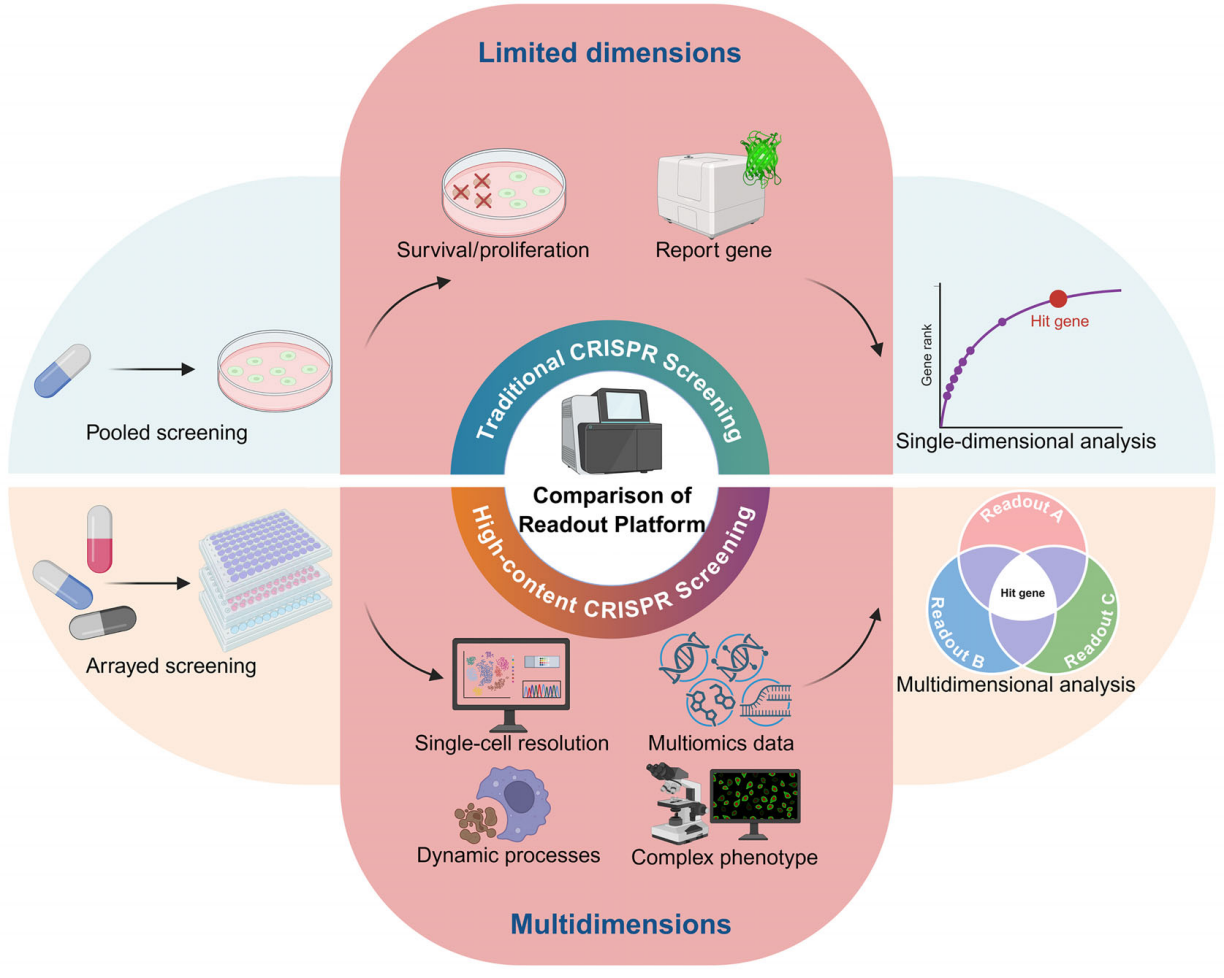
The evolution from traditional to high‐content CRISPR screening. Traditional CRISPR screening based on survival exhibits a relatively straightforward workflow, yet yields limited information due to its singular phenotypic readout dimension (e.g., survival/proliferation). High‐content phenotype‐resolved CRISPR screening achieves multidimensional deep analysis through integration with multimodal profiling technologies. It simultaneously captures multidimensional information including spatial positioning, multiomics data, complex phenotypes, and dynamic biology processes, thereby comprehensively elucidating the relationship between gene function and phenotype. By cross‐analyzing the intersection of these multimodal readouts, this integrated approach comprehensively deciphers the intricate relationships between genetic perturbations and complex phenotypes.

Recent technological advances in single‐cell sequencing, spatial transcriptomics, high‐content imaging, and multiomics profiling have driven the transition from traditional phenotype‐restricted CRISPR screens toward high‐content, phenotype‐resolved CRISPR screening frameworks. By integrating pooled genetic perturbations with multidimensional molecular and imaging readouts, these approaches enable systematic characterization of transcriptional states, proteomic changes, metabolic adaptations, and spatial cellular organization following genetic perturbations [10, 11, 12, 13, 14, 15]. By significantly expanding analytical depth, these developments establish high‐content CRISPR screening as a powerful framework that transcends simple fitness phenotypes to link genetic perturbations to complex cellular phenotypes and biological mechanisms in vitro and in vivo. Figure 1 illustrates the evolution of CRISPR screening platforms from traditional readout strategies to high‐content multimodal profiling approaches.

Although several recent reviews have summarized CRISPR screening in specific research areas such as cancer immunotherapy and therapeutic target discovery [16, 17, 18], a systematic synthesis of the methodological evolution of high‐content CRISPR screening and its cross‐disciplinary applications remains lacking. In this review, we focus on the conceptual and technological transition toward high‐content CRISPR screening, emphasizing advances in perturbation strategies, library design, delivery systems, and multimodal readout platforms. Furthermore, we summarize representative applications of high‐content CRISPR screening across multiple biological and biomedical disciplines, including cancer biology, immunology, developmental biology, neurobiology, and infectious diseases, highlighting how these approaches enable more precise functional annotation of gene regulatory networks. We also discuss the progress in translating CRISPR‐identified targets into clinical investigation. Finally, we examine current technical challenges in the field, including scalability, perturbation fidelity, cost, and limitations of in vivo screening approaches, and discuss future directions involving deeper spatiotemporal phenotyping and multimodal integration. This review aims to provide a comprehensive framework for understanding the methodological landscape and future development of high‐content CRISPR screening.

## 2 Methodology of High‐Content CRISPR Screening

The successful execution of a high‐content CRISPR screening requires the seamless integration of multiple technological modules. This section systematically outlines these core components, beginning with the selection of perturbation modalities and library design, followed by an evaluation and comparison of viral and nonviral delivery systems, focusing on their working principles, technical advantages, key limitations, and applicable scenarios. We then discuss the application of these tools across diverse in vitro and in vivo disease models, concluding with a review of the cutting‐edge high‐content readout platforms that set this approach apart from conventional tools such as RNA interference (RNAi).

### 2.1 CRISPR Perturbation Modalities and Library Design

Although high‐content screening yields diverse phenotypic readouts, the core of the method rests on the diversification of perturbation modalities and the deliberate design of their libraries. For different CRISPR perturbation modalities such as CRISPR knockout (CRISPRko), CRISPR interference (CRISPRi), CRISPR activation (CRISPRa), it is necessary to design guide RNA (gRNA) libraries with specific targeting rules that are compatible with each respective method. Researchers can employ different libraries tailored to specific biological questions, broadly categorized as whole‐genome libraries and targeted libraries.

#### 2.1.1 CRISPR Knockout

CRISPR–Cas9 technology has revolutionized the distribution of genotypes and phenotypes through large‐scale loss‐of‐function (LOF) screening. CRISPRko, as a foundational modality, relies on wild‐type Cas9 protein to induce double‐strand breaks (DSBs) at target sites under gRNA guidance. This subsequently introduces frameshift mutations via the error‐prone nonhomologous end‐joining (NHEJ) repair mechanism, thereby achieving permanent gene loss [19]. The greatest advantage of CRISPRko lies in the permanent and complete nature of its gene knockout, making it suitable for investigating the long‐term effects of genes. However, the limitations of CRISPRko stem precisely from the potency of its gene knockout. CRISPRko irreversibly disrupts genes by inducing DSBs and frameshift mutations, rendering it unsuitable for investigating acute, reversible effects of gene function. Moreover, NHEJ operates with low efficiency in nondividing or inactive dividing cells (such as neurons, cardiomyocytes, and terminally differentiated cells etc.), rendering CRISPRko unsuitable for these nondividing cells [20]. CRISPRko screening must also account for the impact of off‐target effects, namely, unintended cleavage at nontarget genomic sites induced by gRNA‐guided Cas9, which may introduce false positive or false negative signals [21]. This problem can be mitigated through strategies such as optimizing gRNA design [22, 23] and employing high‐fidelity Cas9 variants [24, 25, 26].

#### 2.1.2 CRISPR Interference

CRISPRi employs a catalytically inactive Cas9 (dCas9) to inhibit transcription without cleaving the DNA double strand, thereby suppressing the target gene [27, 28]. Therefore, CRISPRi is also functionally classified as a LOF screening. In CRISPRi screening, gene function can be restored once dCas9 is removed. This reversibility renders it suitable for functional recovery experiments or investigations into the dynamic changes in gene function [29, 30]. As it does not induce DSBs, CRISPRi offers greater safety and lower off‐target effects compared with CRISPRko [28, 31]. The low cytotoxicity and high safety profile of CRISPRi make it widely applicable for researches involving nonlethal cellular phenotypes, including metabolism [32], mitochondrial function [33], and immune signaling pathways [34]. However, CRISPRi‐mediated gene disruption is more sensitive to gRNA selection and often results in incomplete silencing compared with the use of active Cas9 [35]. To address this issue, researchers improve dCas9 and refine the Krüppel‐associated box (KRAB) domain [36], which have partially mitigated the problem. Moreover, Stadager et al. developed an innovative gene‐editing system named CRISPRgenee, which for the first time integrates CRISPRko and CRISPRi technologies into a single system via a dual‐sgRNA vector design, thereby increasing LOF rates without increasing genotoxic stress, facilitating library size reduction for advanced LOF screens [37].

#### 2.1.3 CRISPR Activation

CRISPRa is a gain‐of‐function (GOF) screen approach that functions by binding dCas9 to various transcription activators, thereby elevating the expression levels of target genes [38]. The first‐generation CRISPRa system, designated dCas9–VP64, was constructed by fusing the C‐terminus of dCas9 to four herpes simplex virus‐derived VP16 transcriptional activation domain [39, 40]. This configuration typically exhibits low to moderate efficiency in activating endogenous gene expression. To enhance the efficiency of gene transcriptional activation, Konermann et al. developed the CRISPR Synergistic Activation Mediator system. In this system, an MS2 hairpin aptamer is appended to the sgRNA, which in turn recruits the MS2–p65–HSF1 fusion protein. The combined action of these three distinct activation domains synergistically amplifies transcriptional activation efficiency [38]. Building on the principle of synergistic activation, further innovations have focused on achieving higher efficiency and more compact system architectures. CRISPR VP64–p65–Rta substantially enhances gene activation levels by replacing VP64 with an improved transcriptional regulator that fuses three activation domains: VP64, p65, and Rta [41]. The SunTag protein‐recruitment system has also been utilized to optimize CRISPRa.​ In this approach, multiple copies of the short epitope peptide GCN4 are fused to dCas9, while the transcriptional activator VP64 is fused to its cognate single‐chain variable fragment (scFv) antibody. This design enables the GCN4–dCas9 fusion to efficiently recruit multiple copies of scFv–VP64, thereby amplifying target gene expression [42, 43]. Compared with conventional overexpression techniques, CRISPRa efficiently drives gene expression by activating endogenous promoters, thereby eliminating the need for constructing exogenous expression elements and overcoming limitations related to transcript size. The dCas9‑mediated gene regulation is reversible and does not cause permanent changes in genomic DNA [44]. It can also enable simultaneous regulation of multiple target genes [45]. Moreover, by fusing dCas9 with fluorescent proteins, the localization of target genes can be visually tracked [46, 47, 48]. However, the broad application of CRISPRa is constrained by its oversized components and the potential immunogenicity, necessitating optimized delivery strategies for robust and safe expression [49]. Moreover, as with all other CRISPR–Cas systems, there exists a risk of immune reaction in target cells against bacterial Cas protein [44, 50].

#### 2.1.4 Base Editing and Prime Editing

Base editors are engineered fusion proteins that combine a catalytically impaired Cas nuclease with a deaminase enzyme. Rather than creating DSBs, they directly catalyze the chemical conversion of specific nucleobases on a DNA strand. To date, two primary classes have been developed: cytosine base editors, which enable the conversion of a C•G base pair to T•A, and adenine base editors, which facilitate the conversion of A•T to G•C [51]. As it does not rely on DSBs and the error‐prone NHEJ repair pathway, the probability of generating random insertions/deletions is extremely low. Moreover, there is less adverse events associated with DSBs in base editors [21, 52]. Driven by the pursuit of greater versatility and precision beyond single‐base conversions, prime editing was developed as a “search‐and‐replace” technology. Prime editing is a versatile and precise genome‐editing method that directly writes new genetic information into a specified DNA site. This is achieved by using an engineered fusion protein comprising a Cas9 nickase and a reverse transcriptase, which is programmed by a prime editing guide RNA (pegRNA). The pegRNA both specifies the target genomic locus and encodes the desired edit [53]. The team led by Jesse M. Engreitz developed and systematically validated a high‑content platform named “Variant‑EFFECTS.” This innovative platform integrates CRISPR prime editing with flow‑based RNA fluorescence in situ hybridization or fluorescence antibody detection, enabling the simultaneous introduction and quantitative measurement of the precise effects of hundreds of designed DNA sequence edits on target gene expression in the endogenous genomic context [54]. Base editing, as a precise gene editing tool, has demonstrated significant potential in the field of genetic disease treatment, including duchenne muscular dystrophy, inherited retinal diseases, genetic hearing loss, hematological hereditary diseases, phenylketonuria, familial hypercholesterolemia, dilated cardiomyopathy, and CD3δ severe combined immune deficiency and spinal muscular atrophy [55, 56]. However, base editing still faces challenges such as low efficiency, the need for optimization of delivery methods, and concerns regarding long‐term safety and associated clinical risks [56, 57].

#### 2.1.5 CRISPR Screening Library Design

Based on the distinct features of the aforementioned CRISPR perturbation modalities, the key to translating them into practical screening tools lies in the targeted design of gRNA libraries. Depending on the research objective, gRNA libraries generally fall into three categories distinguished by screening scope and design flexibility. Genome‐wide libraries, such as the widely used Brunello library, enable systematic, unbiased discovery of gene function across the entire genome but require substantial cell numbers and deep sequencing coverage, making them costly and resource‐intensive [58, 59, 60, 61, 62, 63, 64]. Targeted libraries, in contrast, focus on predefined gene sets to investigate genes related to specific biological questions [65, 66, 67]. Furthermore, custom‐designed libraries are employed for complex biological scenarios, allowing researchers to freely design and construct gRNA libraries tailored to highly specific and individualized scientific inquiries, thereby addressing screen goals beyond the scope of standard commercial libraries [68, 69].

Beyond the choice of library type, several universal design principles are critical for screen success. These include employing robust algorithms for selecting highly active sgRNAs with minimal off‐target effects [23]. The coverage depth and complexity of the library are crucial for maintaining statistical power. The library should contain a sufficient number of gRNA to ensure that each gRNA maintains adequate initial coverage depth in infected cells throughout the experiment [70]. The delivery of the CRISPR library must be stringently controlled, as the objective of this step is to achieve highly efficient and uniform introduction of the library into the target cell population. A critical aspect of delivery is the precise determination of the optimal multiplicity of infection (MOI) to ensure that the majority of cells integrate only one gRNA. This is paramount to prevent the confounding of the relationship between the genetic barcode and the resulting phenotypic outcome [70, 71].

### 2.2 Library Delivery System for CRISPR Screening

The application of CRISPR/Cas systems in gene editing and screening relies upon the effective delivery of CRISPR components (Cas proteins and gRNA) into target cells. The choice of delivery system represents a critical consideration in the design of CRISPR screening experiments, as it directly influences editing efficiency, safety, and suitability across diverse models. Here, we review the most widely employed CRISPR/Cas9 delivery systems, which can be broadly categorized into two groups: viral vector delivery and nonviral delivery methods.

#### 2.2.1 Viral Vector Delivery

Viral vectors occupy a significant position in CRISPR delivery due to their innate infectivity and highly efficient gene transduction capabilities [72]. Commonly used viral vectors include lentivirus (LVs) and adeno‐associated virus (AAVs).

A lentiviral vector is a viral vector system based on the HIV‐1 virus with modifications. The packaging capacity of the LV is around 8 kb [73]. LVs, with a diameter of approximately 100 nm, generally consist of several essential components, including the 5′ long terminal repeat (LTR), the Ψ packaging signal, the central polypurine tract/chain termination sequence, the Rev responsive element, and the 3′ LTR, which contains the poly(A) signal [74, 75]. LVs are the predominant delivery vector for pooled CRISPR screens. They enable stable genomic integration of genetic perturbations, ensuring sustained expression of the desired genes without loss during cell division. Moreover, LVs demonstrate high transduction efficiency in both dividing and nondividing cells. To address the challenges in hard‐to‐transduce suspension cells, Napoleone et al. developed a two‐step static transduction protocol based on LVs. This approach enhanced transduction efficiency in suspension‐adapted Chinese hamster ovary‐K1 and human embryonic kidney 293‐6E cells [76].

LVs are primarily applicable for in vitro gene editing, particularly for large‐scale functional genomic screening to investigate gene function, drug targets and disease mechanisms [77, 78, 79, 80, 81, 82]. Exemplified by CROP‐seq, the technology integrates the hU6‐gRNA construct into the 3′ LTR region of the LVs, leveraging viral integration to enable cotranscription with the host genome. Since the gRNA is embedded within a polyadenylated mRNA transcript, its sequence can be directly detected via single‐cell RNA sequencing (scRNA‐seq). This vector architecture establishes a direct link between genetic perturbations and single‐cell phenotypes without requiring additional barcoding, thereby significantly enhancing both the accuracy and throughput of screening [15].

Despite these advantages, LVs have several shortages represented by the risk of insertional mutagenesis. LVs randomly integrate their carried sgRNAs into the host genome, a process that may disrupt tumor suppressor genes, activate oncogenes, or interfere with endogenous regulatory elements like promoters and enhancers, thereby introducing phenotypic artefacts unrelated to the targeted gene disruption [83]. The particle components and contaminants of LVs can trigger immune responses. This nonspecific immune activation severely interferes with the accurate assessment of physiological processes such as the TME, leading to biased screening results [84]. While the packaging capacity of lentiviral vectors (typically 8–10 kb) is generally sufficient for standard CRISPRko constructs, it is often limiting for delivering larger payloads such as bulkier Cas variants or more complex regulatory elements [73, 74].

AAVs are nonenveloped, single‐stranded DNA virus composed of an icosahedral protein capsid of ∼26 nm in diameter and a single‐stranded DNA genome of ∼4.7 kb that can either be the plus (sense) or minus (antisense) strand [85]. Its most significant advantages over LVs are its minimal immunogenicity and favorable safety profile, which render it better suited for in vivo CRISPR screening applications [86]. Leveraging these advantages, recent studies have successfully implemented AAV‐based high‐content screening to dissect complex biological processes in vivo. Zheng et al. utilized massively parallel AAV‐mediated in vivo Perturb‐seq to map cell‐type‐specific transcriptional networks during tissue development, demonstrating the capability of AAVs to capture dynamic gene regulatory changes in a living organism [87]. Santinha et al. applied AAV‐mediated direct in vivo single‐cell CRISPR screening (AAV‐Perturb‐seq) using gene editing and transcriptional inhibition to systematically dissect the phenotypic landscape underlying 22q11.2 deletion syndrome [3, 4] genes in the adult mouse brain prefrontal cortex [88]. Peng et al. performed unbiased functional mapping of tumor‐infiltrating natural killer cells using in vivo AAV–Sleeping Beauty–CRISPR screens in solid tumor mouse models to identify genetic checkpoints of chimeric antigen receptor (CAR‐NK) therapy [89]. Kampmann's team developed a new in vivo screening platform named CrAAVe‐seq, that employs AAV‐delivered Cre‐dependent sgRNA libraries coupled with episome sequencing to enable high‐content, low‐cost, and highly sensitive CRISPRi screening in specific cell types in the mouse brain [90]. These applications underscore AAVs as a robust and versatile tool for high‐content phenotyping in intact organisms. However, the application of AAVs in CRISPR screening is constrained by its limited packaging capacity (less than 5.2 kb) [91], which often necessitates the use of dual‐vector systems and Cas variants [92, 93], thereby increasing experimental complexity and potentially restricting the targeting scope. Due to its persistence primarily as episomal forms without integration into the host genome, AAV‐delivered transgene expression is rapidly diluted in rapidly dividing cells as proliferation proceeds [94]. This inherent limitation makes AAVs fundamentally unsuitable for conventional, long‐term proliferation‐based in vitro pooled screening approaches, and thus it is better suited for applications involving nondividing cells or short‐term phenotypic screens.

#### 2.2.2 Nonviral Vector Delivery

Although viral vectors are extensively employed in screening applications, their complex production processes, potential immunogenicity, and limited payload capacity constrain their application in high‐content screening. Nonviral delivery methods, owing to their high safety and low immunogenicity, have emerged as important supplements or alternatives to viral vectors such as LVs and AAVs.

Ribonucleoprotein (RNP) delivery, the predominant nonviral delivery method, refers to the in vitro assembly of the Cas9 protein with gRNA into an RNP complex for direct introduction into cells. In contrast to DNA‐based vectors that rely on the host cell's transcription and translation machinery, the RNP complex exhibits immediate nuclease activity upon cellular entry without requiring prior expression, thereby enabling rapid and precise gene knockout [95]. The RNP complex operates with a remarkably short functional duration. After completing the editing process, it is swiftly degraded by cellular proteases, embodying a distinctive “fast on, fast off” operational strategy [25].

RNP delivery has been extensively employed in high‐content CRISPR screening. For example, the high‐content RNP screening platform established by Hultquist et al. enabled the systematic dissection of HIV host dependency factors in primary T cells [96]. Meier et al. transfected RNPs into induced pluripotent stem cell (iPSC)‐derived myeloid cells, identifying the mammalian target of rapamycin complex 1 (mTORC1) signaling pathway as a critical modulator of lipid storage in both *APOE3* and *APOE* knockout microglia [97]. The CRISPR‐READI (CRISPR RNP Electroporation and AAV Donor Infection) technology, developed by Chen et al., leverages the synergy between Cas9/sgRNA RNP electroporation and AAV donor infection to establish an efficient, high‐throughput, and microinjection‐free platform for sophisticated genome engineering in mice, with promising potential for extension to other mammalian species [98]. Although RNP delivery offers significant advantages in arrayed screening, its limitations must not be overlooked. The rapid intracellular degradation of RNP complexes may constrain their utility in research requiring sustained phenotypic observation [99]. Additionally, the production or procurement of RNP components is costly, and they are susceptible to contamination by toxic substances [100, 101]. The reliance on electroporation for RNP delivery induces cytotoxicity and cellular stress, which may confound sensitive phenotypic readouts in high‐content screening [102]. This physical approach is also largely restricted to in vitro settings. To address these limitations, lipid nanoparticle (LNP) has emerged as a versatile nonviral delivery system.

LNPs are currently the most clinically successful nonviral nucleic acid delivery system with four primary components: ionizable cationic lipids, polyethylene glycol (PEG) lipids, zwitterionic phospholipids, and cholesterol [103]. Ionizable cationic lipids have positively charged headgroups that can encapsulate nucleic acids through electrostatic interactions [104]. PEG lipids, with their hydrophobic tails inserted into the lipid layer of LNPs, form a hydrophilic steric barrier on the surface to prevent aggregation [105, 106]. Phospholipids and cholesterol, regarded as auxiliary lipids that provide stability, interact to maintain the lipid bilayer in an ordered phase, thereby preserving its integrity [107]. Similar to RNPs, LNPs also demonstrate favorable biosafety profiles characterized by low off‐target effects and nonintegration properties. However, unlike RNPs, which is constrained by electroporation‐induced cytotoxicity, LNPs are safer and gentler on cells. Because it is well tolerated and gets into cells through an endocytic process, it keeps cells healthy and is an ideal delivery vector for high‐content studies [108, 109]. Rouatbi et al. developed an LNP platform capable of efficiently codelivering Cas9 mRNA and gRNA, which achieved in vivo gene editing in an orthotopic model of mesenchymal glioblastoma cancer stem cells, demonstrating the potential of LNPs as vector for in vivo delivery [110]. It is noteworthy that Chen et al. developed a delivery platform utilizing LNP encapsulation and delivery of RNPs, enhancing editing efficiency while integrating the advantages of both delivery methods [111]. While LNPs overcome the cytotoxicity associated with electroporation dependent RNP delivery by utilizing a gentler endocytic pathway, they face challenges similar to RNPs as nonviral vectors. LNP gene editing does not integrate into the genome and cannot achieve stable heredity as lentiviral vectors do. Consequently, the editing effect or exogenous material is rapidly diluted with cell division. This makes LNPs difficult to apply in CRISPR screens that require long‐term tracking of phenotypic changes or use cell proliferation as a readout. Additionally, the ionizable lipid component of LNPs may elicit immune responses [112], activating multiple inflammatory pathways and inducing interleukin (IL)‐1β and IL‐6 [113]. Moreover, the manufacturing process for LNPs is more complex and incurs higher production costs.

Beyond conventional RNPs and LNP, a new generation of stimuli‐responsive polymeric delivery vectors has been engineered to achieve spatiotemporally controlled delivery with potential use in high‐content CRISPR screening [114]. Microfluidic cell deformation is an emerging physical delivery technology that facilitates efficient intracellular delivery of macromolecules by inducing transient deformation of the cell membrane, demonstrating promise for applications in CRISPR screening [115, 116]. Cell‐penetrating peptides (CPPs) are short, membrane‐translocating peptides capable of delivering macromolecular cargoes, such as RNPs, into cells via noncovalent complexation, offering a transient, low‐toxicity, and nonviral alternative to traditional vector systems [117, 118]. Exosomes, ranging in size from approximately 40 to 150 nm, are extracellular vesicles of endosomal origin that are secreted by most cell types [119]. Capable of carrying and transferring diverse biomolecules including plasmids, proteins, small interfering RNA (siRNA), and microRNA (miRNA) to recipient cells, exosomes have emerged as an efficient delivery vehicle for CRISPR/Cas9 component, holding promise to overcome the major limitations associated with viral and other nonviral delivery vectors [120, 121]. Xiong et al. developed a recombinase‐based method utilizing Bxb1 recombinase mediated cassette exchange as delivery vector for virus‐free, genome‐wide CRISPRko screens [122].

**FIGURE 2:**
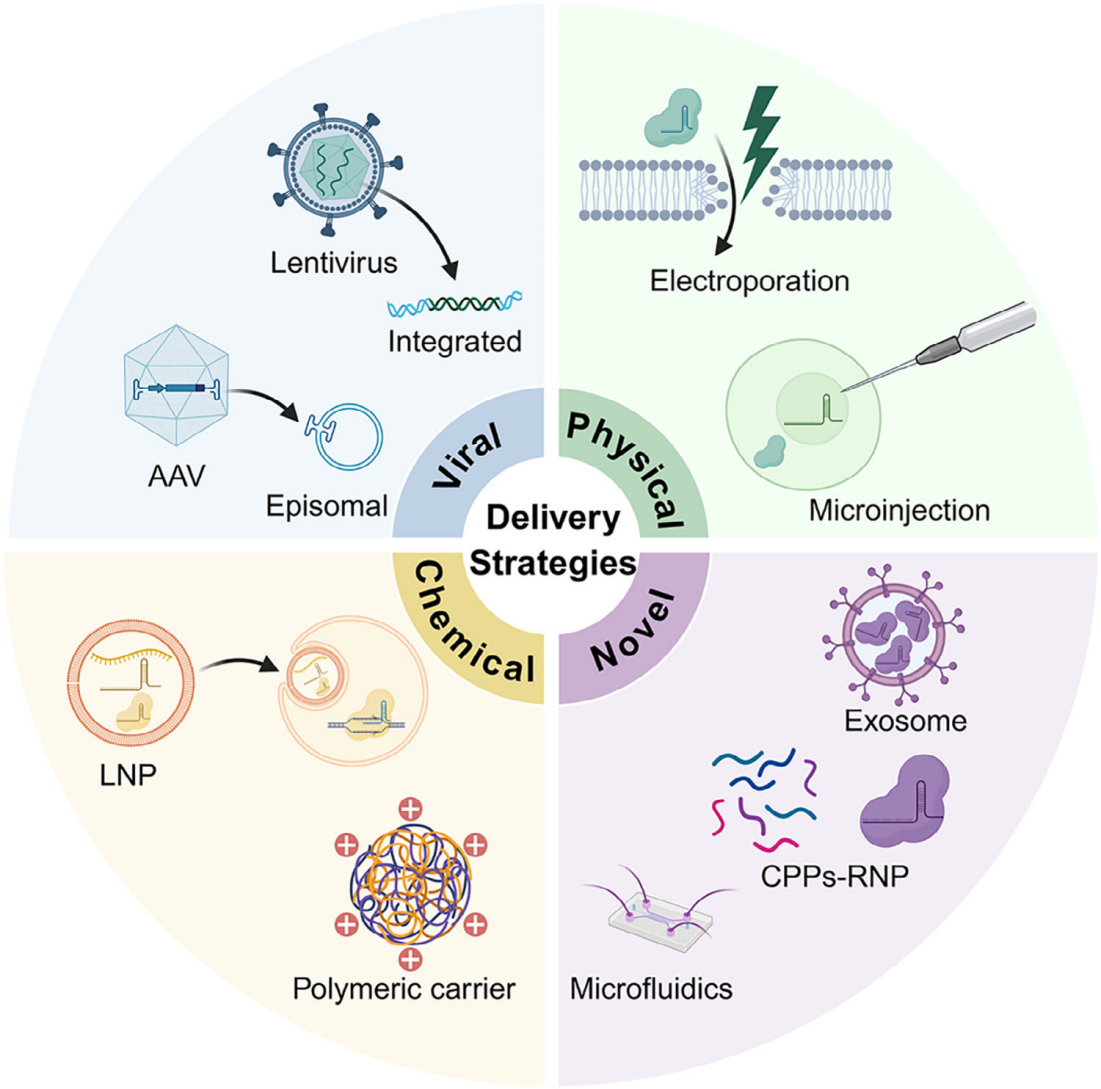
Summary of delivery strategies in high‐content CRISPR screening. Viral vector delivery: primarily represented by LVs and AAVs. LVs are the most widely used delivery vector enabling genomic integration and long‐term stable expression of CRISPR components. AAVs, known for their low immunogenicity and nonintegrating properties, serve as important tools for in vivo screening and primary cell applications; physical delivery: includes methods such as electroporation and microinjection, which allow efficient direct intracellular delivery of RNPs or nucleic acids, particularly suitable for hard‐to‐transfect cells; chemical delivery methods: mainly comprise novel chemical carriers like LNPs and polymeric carriers, which can efficiently encapsulate and deliver nucleic acids or RNP, serving as crucial tools for high‐throughput screening and in vivo delivery; novel vector delivery: represented by exosomes and CPPs, this field is rapidly advancing, aiming to enhance delivery efficiency, cell targeting, and in vivo application potential.

In summary, the delivery system represents a decisive factor in the design and success of high‐content CRISPR screening. The primary delivery systems are illustrated in Figure 2 and Table 1. While viral vectors remain indispensable for stable, long‐term in vivo applications, nonviral RNP and LNP platforms offer safer, transient alternatives, requiring researchers to carefully balance delivery efficiency and integration risks against the specific temporal demands of their screening models.

**TABLE 1:** Comparison of CRISPR delivery systems: characteristics, advantages, limitations, and optimal applicable scenarios.

Evaluation aspects	LV [73, 74, 75]	AAV [85, 86, 91, 94]	RNP [25, 95, 99, 100, 101]	LNP [103, 108, 109, 112, 113]	Physical delivery [115, 116]	Novel delivery (exosome/CPPS/microfluidics) [117, 118, 119, 120, 121, 122]
Key mechanism	Integrate into host genome	Persist as nuclear episomes	Act as preassembled complexes	Deliver payloads via lipid encapsulation	Permeabilize membranes via physical forces	Traverse membranes via noncanonical pathways
Advantages	High transduction efficiency; stable, long‐term expression	Nonintegrating (episomal state); good safety profile	Fast action, short duration; high precision and low off‐target effects	Good safety and versatility; high precision and low off‐target effects	Direct and efficient; ideal for hard‐to‐transfect cells	High biocompatibility and low immunogenicity; high potential for in vivo applications
Limitations	Risk of insertional mutagenesis and genomic disruption; immunogenicity limits in vivo use	Episomal persistence leads to rapid dilution in dividing cells; unsuitable for long‐term pooled screens	Electroporation‐induced cytotoxicity and cellular stress; rapid intracellular degradation	Potential lipid‐induced inflammatory responses; complex and costly manufacturing	High cytotoxicity and cellular stress; restricted to in vitro use	Largely in exploratory phase
Optimal applicable scenarios	Large‐scale in vitro pooled screens; stable genomic integration required	In vivo CRISPR screening; nondividing cells or short‐term screening	Rapid and precise gene knockout; arrayed CRISPR screening	In vivo CRISPR screening; short‐term or sensitive phenotypic studies	Hard‐to‐transfect suspension cells	Virus‐free CRISPR screening

Abbreviations: AAV, adeno‐associated virus; LNP, lipid nanoparticle; LV, lentivirus; RNP, ribonucleoprotein.

### 2.3 Cellular and Disease Models for CRISPR Screening

#### 2.3.1 In Vitro CRISPR Screening

In vitro CRISPR screening, centered on two‐dimensional (2D) cell lines, serves as a foundational platform for functional genomics. Its core purpose is to enable rapid, large‐scale, and low‐cost initial gene function screening, primarily for investigating fundamental biological processes such as cell proliferation, survival, and drug sensitivity. As the most mature and widely applied screening format, in vitro 2D cell line screening establishes a critical foundation for subsequent validation in more complex models, such as organoids or in vivo systems.

In 2014, a landmark study was published by the Zhang et al., definitively establishing the feasibility of genome‐wide CRISPRko screening [19]. In this work, genome‐scale functional interrogation was accomplished through the delivery of a LV CRISPR library into immortalized cancer cell lines, followed by the application of pharmacological agents or toxin‐mediated challenges to exert selective pressure and facilitate the enrichment of resistant clones. Subsequent deep sequencing analysis enabled the identification of genes, including NF2, CUL3, TADA2B, and TADA1. Since then, genome‐wide in vitro CRISPR screening, leveraged for its unique capacity for unbiased interrogation, has been instrumental in elucidating key regulatory elements across diverse fields, including immunology, cancer biology, and stem cell research.

While screening in immortalized cell lines offers notable advantages in efficiency and experimental convenience, their prolonged culture and transformed nature often result in accumulated genetic aberrations and the loss of certain native characteristics. To overcome this bottleneck, efforts have been directed toward establishing CRISPR screening platforms directly in primary cells. Through optimized electroporation protocols and refined gRNA library designs, several groups including Marson et al. have successfully conducted genome‐wide screens in primary human T cells, leading to the identification of previously uncharacterized genes governing T cell activation and exhaustion [123, 124, 125, 126]. Beyond T cells, the application of CRISPR screening technologies has been extended to a range of primary innate immune cells, including dendritic cells (DCs), macrophages, and NK cells. Genome‐wide interrogation in these cell types has uncovered novel regulators of phagocytosis, inflammatory signaling, and antigen presentation [127, 128, 129, 130]. Collectively, these advances underscore the transformative potential of unbiased functional screening in primary cell systems.

#### 2.3.2 In Vivo CRISPR Screening

Notwithstanding the advantages and insights gained from in vitro cellular models, such systems possess intrinsic limitations when employed to recapitulate complex biological processes in vivo. Consequently, the metabolic and differentiation states of cultured cells often diverge substantially from their in vivo counterparts [131, 132]. To transcend these limitations, efforts have been directed toward adapting CRISPR screening platforms for in vivo applications. In oncology, this endeavor was pioneered through indirect transplantation‐based in vivo screens. This approach involves ex vivo infection of cancer cell lines with a pooled gRNA library, followed by engraftment of the mutagenized population into immunocompromised mice. After tumor progression or metastatic dissemination, gRNA enrichment patterns within primary and secondary lesions are interrogated via deep sequencing to pinpoint genes governing tumor growth and metastasis. The feasibility of genome‐scale in vivo CRISPR screening was first demonstrated by Chen et al. in 2015, whose work in murine tumor models identified *Nf2*, *PTEN*, and *Cdkn2a* as critical regulators of tumor progression and pulmonary metastasis [133].

While these transplantation‐based models have yielded valuable insights, they fail to recapitulate the entirety of neoplastic evolution as it unfolds from normal cells in their native tissue context. This limitation has driven the development of direct in vivo CRISPR screening, where gRNA libraries are delivered directly into target organs of Cas9 transgenic animal models. Leveraging an AAV–SB–CRISPR hybrid vector platform, Chen et al. have conducted seminal work enabling high‐throughput in vivo genetic screening within immune compartments. In 2019, this system was first deployed in primary CD8^+^ T cells, achieving efficient gene editing and enabling genome‐scale in vivo interrogation. Analysis of three independent in vivo screens in a glioblastoma model identified *Pdia3*, *Mgat5*, and *Emp1* as candidate membrane protein‐encoding genes [134]. Building upon this foundation, the group extended their screening platform to primary NK cells in 2024, accomplishing the first large‐scale, unbiased genetic screening in primary NK cells in vivo. Integrative analysis of screening hits with scRNA‐seq profiles of tumor‐infiltrating NK cells revealed that ablation of calcium homeostasis modulator 2 (CALHM2) markedly augmented NK cell and CAR‐NK cell cytotoxicity, degranulation capacity, cytokine secretion, and tumor infiltration [135].

#### 2.3.3 CRISPR Screening in Organoid

Compared with in vivo models, organoids permit the application of well‐defined and uniform selective pressures under controlled culture conditions while circumventing the complex systemic interference inherent to living organisms. Relative to 2D in vitro systems, human organoids retain tissue‐specific cellular composition and 3D architecture, enabling direct interrogation of human‐specific mechanisms and facilitating temporally resolved functional screens [136, 137]. The development of organoid models can be clearly illustrated in neuroscience. Early 3D cultures, such as neurospheres, provided foundational platforms for investigating neural stem cell (NSC) proliferation and early differentiation [138]. Subsequent advances enabled the derivation of human iPSC‐derived brain organoids. For instance, a mechanical injury model incorporating genome‐wide CRISPRi screening identified the potassium channel *KCNJ2* as a critical mediator of neuronal death and TDP‐43 pathology, thereby uncovering potential therapeutic targets for traumatic brain injury [139]. A major methodological milestone was achieved with the advent of assembloid technology, which allows region‐specific organoids, such as those recapitulating ventral and dorsal telencephalon to be fused, thereby modeling long‐range inhibitory neuron migration. Leveraging this sophisticated system, Pasca et al. conducted a landmark CRISPR screening interrogating 425 neurodevelopmental disorder (NDD) risk genes, identifying 33 regulators of neuronal migration and revealing a previously unrecognized role for *LNPK* in modulating endoplasmic reticulum localization during this process [6, 140]. Concurrently, organoids have been extensively deployed for multidimensional CRISPR screening across diverse cancer types, facilitating the discovery of both established and novel therapeutic targets. An in vivo CRISPR screening utilizing mouse gastric organoids not only validated the central roles of PTEN/Akt and transforming growth factor β (TGF‐β) signaling in gastric carcinogenesis but also uncovered a mechanism by which Helicobacter pylori promote tumor growth through recruitment of SiglecF^+^ neutrophils [141]. In human gastric cancer (GC) organoids, a comprehensive screening platform integrating CRISPR knockout, interference, and activation modalities with scRNA‐seq readouts was established to systematically delineate the molecular networks underlying cisplatin resistance; this approach identified *TAF6L* and the GMDS‐fucosylation axis as novel therapeutic vulnerabilities [29]. In colorectal cancer research, patient‐derived organoids were combined with single‐cell sequencing and transcription factor‐focused CRISPR screening to reveal that cells escape KRAS/EGFR‐targeted therapy through lineage plasticity toward a Paneth‐like state, a process driven by SMAD1–FGFR3 signaling, with inhibition of this axis resensitizing tumors to treatment [142]. *CDK12* was established as a bona fide tumor suppressor in prostate cancer through integrated organoid culture and CRISPR screening approaches. Its loss was found to induce genomic instability via transcription–replication conflicts and, notably, to confer synthetic lethality with its paralog *CDK13*, thereby defining a precision therapeutic strategy for this aggressive subtype [143].

Despite substantial progress in recapitulating tissue complexity and disease mechanisms, current organoid models incompletely capture the cellular diversity of human organs, particularly lacking key components such as immune and endothelial cells. Efforts to address this limitation have focused on developing coculture systems that more faithfully model the intricate cellular interactions within native tissues [144]. The neuron–tumor organoid coculture model represents a paradigm of this approach, wherein CRISPR screening was employed to demonstrate that enteric neurons modulate drug sensitivity in GC cells through regulation of tumor cell lipid metabolism, leading to the identification of microenvironment‐dependent vulnerabilities including acetyl‐coenzyme A carboxylase alpha and lanosterol synthase [145]. These targets were entirely undetectable in conventional 2D or monoculture 3D systems (as summarized in Table 2).

**TABLE 2:** Summary of CRISPR screening studies based on organoid models.

Organoid type	Model	Perturbation	Library	Readout	References
Gastrointestinal organoids	Mouse gastric organoids	CRISPR KO	A custom library targeting 49 putative gastric tumor suppressor genes; a “cancer genome‐wide” library targeting 5000 genes	Organoid growth and tumorigenicity in vivo	Jiazhuo He [141]
Gastrointestinal organoids	TP53/APC double knockout (DKO) human gastric cancer organoids	CRISPRi/a; CRISPR KO	CRISPR‐KO library: targeting 1093 membrane protein genes; CRISPRi/CRISPRa library: targeting 1952 DNA/RNA‐binding protein genes	gRNA abundance under cisplatin treatment	Yuanhung Lo [29]
Gastrointestinal organoids	GX117–T1O gastric cancer organoid model	CRISPR KO	Whole‐genome library	Cell proliferation (dropout)	Becky K. C. Chan [145]
Gastrointestinal organoids	Adult intestinal organoids	CRISPR KO	Transcription factor library (targeting 1800 transcription factors)	FACS sorting based on differentiation reporter, gRNA sequencing	Lin Lin [146]
Gastrointestinal organoids	Stem cell‐enriched organoids expressing Cas9 (intestinal organoids)	CRISPR KO	The library contained a total of 4765 sgRNAs	Cell dropout	Sofia Ramalho [147]
Gastrointestinal organoids	3D tumor spheroid model of the human KRAS G12D‐mutant colorectal cancer cell line LS174T	CRISPR KO	Complete Human Transcription Factor CRISPRko Library	Sensitivity to combined *KRAS* and *EGFR* therapy	Yuetong Zhang [142]
Brain organoids	Assembloids	CRISPR KO	Interneuron migration CRISPR sgRNA library (425 NDD genes and 13 genes associated with interneuron development)	Changes in the relative enrichment of sgRNA within specific cell populations	Xiangling Meng [140]
Brain organoids	Mechanical injury model in brain organoids derived from iPSCs; NGN2‐induced cortical spheroids (NGN2‐spheroids)	CRISPRi	Whole‐genome CRISPRi library	Quantification of sgRNA abundance using next‐generation sequencing	Jesse D. Lai [139]
Kidney organoids	Mouse/human kidney organoids	CRISPR KO	Whole‐genome library	gRNA enrichment in self‐renewing versus differentiated NPCs	Biao Huang [148]
Breast cancer organoids	Breast cancer organoids stably expressing Cas9	CRISPR KO	A gRNA library targeting 482 kinase functional domains	Organoid survival and sensitivity to drug combinations	Florencia P. Madorsky Rowdo [149]
Breast cancer organoids	Organoid (CDO) models derived from circulating tumor cells (CTCs) in metastatic breast cancer	CRISPRa	Whole‐genome CRISPRa library	Cell survival under pharmacological stress	Roberto Würth [150]
Prostate organoids	Cdk12 knockout organoids derived from mouse prostate basal cells	CRISPR KO	Mouse whole‐genome cancer gene library (4922 genes)	Organoid growth and sensitivity to *CDK13* inhibition	Jean Ching‐Yi Tien [143]
Prostate organoids	Mouse Pten/Trp53/Rb1 triple‐knockout organoids	CRISPR KO	Customized human NEPC gene‐upregulated sgRNA library (881 genes significantly upregulated in NEPC)	Neuroendocrine differentiation (phenotype and reporter gene)	Hanling Wang [151]
Liver organoids	H813Torg liver cancer organoids resistant to Lenvatinib	CRISPR KO	Whole‐genome library	Cell survival, resistance to Lenvatinib	Yue Guo [152]
Pancreatic organoids	3D organoids derived from primary human pancreatic acinar cells	CRISPR KO	Customized hybrid CRISPR‐KO‐sgRNA lentiviral library	Cellular adaptability, malignant transformation	Yi Xu [69]
Esophageal organoids	Primary mouse esophageal keratinocytes (3D epithelioid)	CRISPR KO	Customized lentiviral gRNA library (targeting the PI3K–mTOR pathway)	Cellular adaptability (growth under nutrient‐limited conditions)	Albert Herms [153]
Esophageal organoids	Esophageal epithelioid cultures	CRISPR KO	Known genes driving clonal expansion; candidate driver genes for esophageal cancer; essential genes	Cloning and expansion capacity (competitive growth)	Albert Herms [154]

This table systematically summarizes representative CRISPR screening studies utilizing diverse organoid platforms. To highlight the tissue‐specific dependencies and distinct microenvironmental contexts inherent to different biological systems, the listed studies are categorized and ordered by their respective physiological organ systems.

Critically, these findings underscore the importance of incorporating microenvironmental components into screening platforms, thereby expanding the repertoire of physiologically and clinically relevant dependencies accessible for therapeutic intervention.

### 2.4 High‐Content Readout Platforms

**FIGURE 3:**
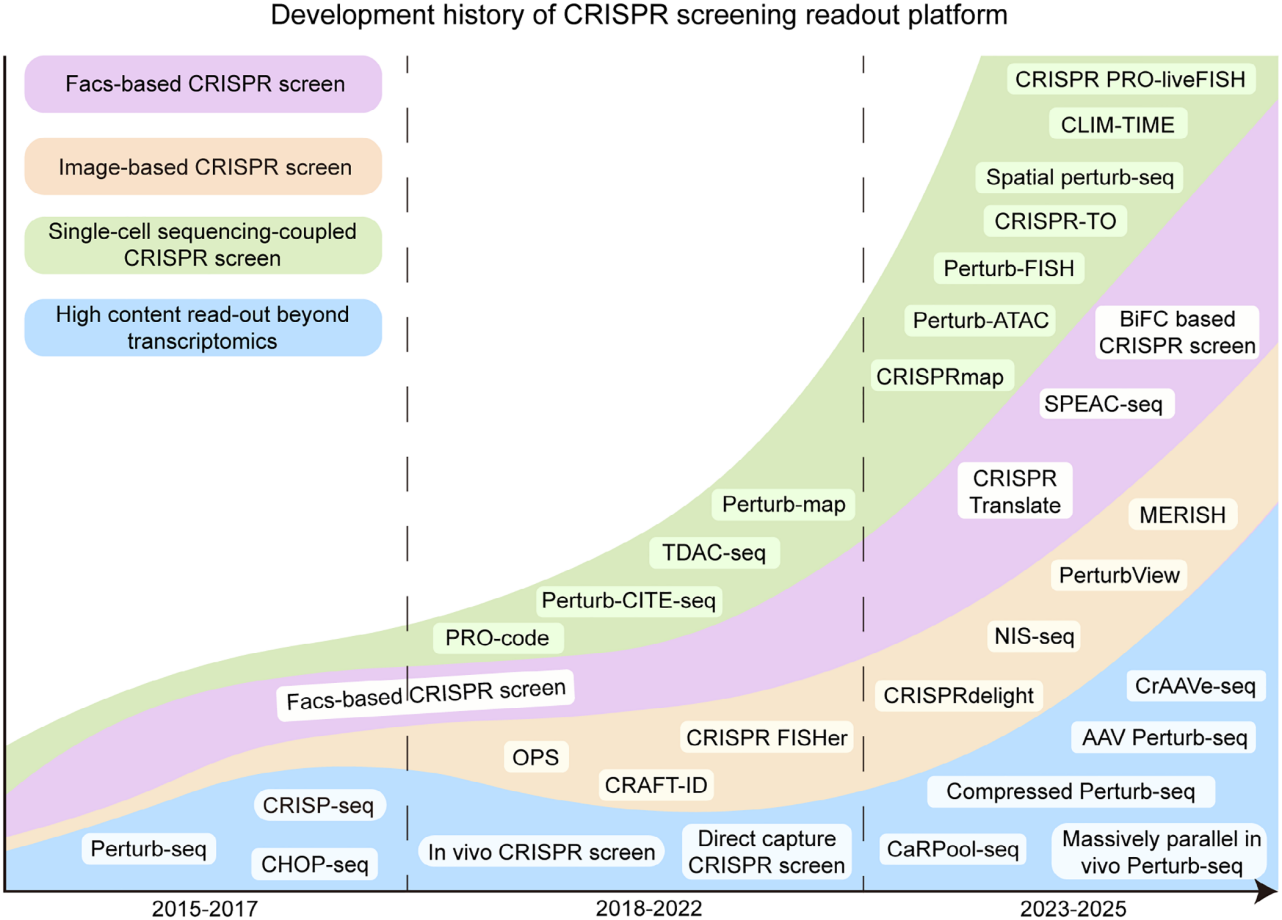
Development history of CRISPR screening readout platform. This shift from early, simple readouts to recent, complex multimodal platforms that integrate single‐cell sequencing, imaging, and spatial data enables the transition from bulk, population‐level analysis to high‐resolution, phenotype‐resolved functional genomics.

CRISPR screening has emerged as an indispensable tool in functional genomics, with its core principle residing in the systematic delineation of causal relationships between genotype and phenotype through large‐scale genetic perturbation coupled with specific readout modalities. In recent years, propelled by rapid advances in imaging technologies, flow cytometry, scRNA‐seq, and spatial transcriptomics, CRISPR screening is progressively transitioning from conventional proliferation‐based survival assays toward a new era of high‐content analysis (Figure 3).

#### 2.4.1 Flow Cytometry‐Based High‐Content CRISPR Screening

Fluorescence‐activated cell sorting (FACS)‐based CRISPR screening is a pooled genetic screening method that combines CRISPR‐mediated gene perturbation with FACS to isolate cells according to a measurable phenotype, thereby enabling the identification of genes that drive that phenotype. Compared with conventional survival‐based screens, the principal advantage of FACS‐based screening lies in its capacity to interrogate phenotypes that cannot be directly translated into proliferative advantage, including cell surface marker expression levels and signaling pathway activity.

In 2015, Stanford University and the Weizmann Institute of Science employed FACS‐based genome‐wide CRISPR screening to identify regulators of the tumor necrosis factor (TNF) signaling pathway in DCs. The investigators delivered an sgRNA library targeting 21,786 protein‐coding genes and miRNA genes, which comprised 125,793 unique sgRNAs. Through FACS‐mediated sorting of TNF‐high and TNF‐low cell populations, this study resolved three functional modules including the TLR pathway, the OST complex, and the PAF complex, and uncovered that the OST complex controls inflammatory responses through endoplasmic reticulum stress and glycosylation [127]. In parallel, recent FACS‐based genome‐wide CRISPR screens have enabled the identification of additional regulators, including *WDFY3*, *FOXH1*, and the cooperative role of *PACT* and *ADAR1* in immune modulation [128, 155, 156]. However, this screening remains poorly suited for capturing complex cellular phenotypes that are not reflected by surface marker expression or bulk fluorescence intensity. Consequently, FACS‐based screening often serves as a complementary initial screening approach within broader high‐content screening pipelines.

#### 2.4.2 Image‐Based High‐Content CRISPR Screening

Before the widespread adoption of high‐throughput CRISPR screening technologies, the conceptual framework for microscopy‐based high‐content screening had already matured. The unique value of image‐based screening, compared with flow cytometric approaches, resides in its capacity to capture spatial information and morphological features within cells. Phenotypes such as cell cycle defects, subcellular localization of proteins or RNAs within organelles, screening in cell types not amenable to flow cytometry, and protein colocalization or aggregation cannot be directly measured by flow cytometry, yet these very phenotypes represent critical dimensions for understanding gene function [157, 158]. In 2019, Feldman et al. developed optical pooled screening, a technology that integrates high‐content imaging with in situ sequencing, enabling direct linkage of genetic perturbations to complex phenotypes at single‐cell resolution. A targeted screen was conducted against the NF‐κB signaling pathway in multiple human cell lines, including HeLa and A549, by monitoring p65 nuclear translocation dynamics. This platform not only recovered known positive and negative regulators such as *MAP3K7*, *CHUK*, but also revealed that mediator complex subunits *MED12* and *MED24* negatively regulate p65 nuclear retention time, thereby modulating downstream chemokine and IL‐1β expression [159]. Subsequently, platforms such as CRaft‐ID and optical enrichment screening based on photoactivatable fluorescent proteins were developed, leading to the identification of multiple regulators including *SNRNP200*, *PUF60*, *KIF18A*, and *NUP62* [160, 161].

Nevertheless, the susceptibility of certain primary cells to processing‐induced damage limits the application of these in situ sequencing methods, as permeabilization often compromises cell viability. In 2024, the Schmid‐Burgk laboratory pioneered the use of NIS‐Seq technology to achieve genome‐wide optical screening across multiple cell types cultured at high density, thereby overcoming the long‐standing limitation of applying optical screens to sensitive cell types [162]. By incorporating a reverse T7 promoter downstream of the gRNA expression cassette, this approach enables in vitro transcription to generate RNA copies within the nucleus for in situ sequencing, thus achieving barcode identification independently of cellular transcriptional activity or intact cell boundaries. As technologies for gRNA lineage tracing continue to mature, the readout modalities of image‐based screening have begun to expand from protein‐level phenotypes toward interrogation of RNA spatial localization. In 2025, a MERFISH‐based imaging screening was reported focusing on the regulation of lncRNA nuclear localization, with MALAT1, which localizes to nuclear speckles and regulates gene expression through alternative splicing, transcriptional control, and miRNA sequestration, as the primary target. Through optimized barcode design, the mismatch rate was reduced to approximately 8%, enabling successful identification of both positive and negative regulators of MALAT1 localization to nuclear speckles [163].

#### 2.4.3 Single‐Cell Sequencing‐Coupled CRISPR Screening

Single‐cell CRISPR screening emerged in 2016, when two concurrent milestone papers published that December collectively established the foundational paradigm of Perturb‐seq. The team led by Aviv Regev and Jonathan Weissman pioneered Perturb‐seq, achieving the first integration of pooled CRISPR screening with scRNA‐seq. By incorporating barcode sequences uniquely matching each gRNA within LV vectors, this technology circumvented the inherent limitation that gRNA lack poly‐A tails and thus evade capture by conventional single‐cell sequencing methods. This enabled the simultaneous readout of genetic perturbations and their resultant transcriptomic changes at single‐cell resolution. The researchers applied it to screen immune‐related transcription factors in DCs and successfully deconvoluting the complex regulatory networks that govern the response of DCs to lipopolysaccharide [12].

While Perturb‐seq proved highly effective in cultured cells, extending this capability to intact organisms was necessary to study gene functions within complex physiological environments. Concurrently, the CRISP‐seq technology developed by Amit's team, built upon a similar conceptual framework, demonstrated the in vivo feasibility of this approach for the first time [14]. In contrast, CROP‐seq does not rely on barcodes to label gRNA, with its core innovation residing in an ingenious vector design that inserts the entire U6 promoter–sgRNA cassette into the 3′ long terminal repeat of the lentiviral backbone. This design caused the gRNA sequence to become unintentionally incorporated into a polyadenylated mRNA transcript upon reverse transcription and integration, rendering it directly accessible to single‐cell sequencing platforms. This design obviated the need to introduce exogenous barcodes for each sgRNA, substantially streamlining library preparation workflows and significantly enhancing the accessibility and adoptability of the technology [15]. Utilizing CROP‐seq, the researchers reconstructed the T cell receptor signaling pathway in Jurkat cells, accurately identifying known key genes such as *LCK* and *ZAP70*, thereby validating the technique's robustness and efficiency.

However, early iterations of three platforms were primarily suited for single‐gene perturbations and susceptible to approximately 50% swapping of gRNA‐barcode associations because of lentiviral template switching. To mitigate the swapping issue, Hill et al. further coupled targeted sgRNA amplification with CROP‐seq, doubling the proportion of cells in which guides are assigned to 94% [164]. In 2020, Replogle et al. developed direct‐capture Perturb‐seq, achieving a technological advancement by employing a 5′‐end sequencing strategy that enabled simultaneous capture of sgRNAs and single‐cell transcriptomes without requiring any modification to the CRISPR library backbone [165]. More importantly, direct‐capture Perturb‐seq facilitated the delivery of multiple sgRNAs per cell, making it possible to investigate genetic interactions and combinatorial perturbation effects at the single‐cell level. The researchers applied this technique to map genetic interaction landscapes between cholesterol synthesis and DNA repair pathways. Further, CaRPool‐seq emerged, pioneering an RNA‐targeting perturbation paradigm by leveraging the CRISPR–Cas13d system [166]. Cas13d enables gene knockdown at the RNA level, offering transient and regulatable effects while avoiding the potential risks associated with DNA DSBs. Through the design of cleavable CRISPR arrays, CaRPool‐seq efficiently achieved multigene RNA perturbations in single cells and uncovered extensive interactions among chromatin regulators during acute myeloid leukemia differentiation.

As screening scales expanded, cost emerged as a major bottleneck. In 2023, compressed Perturb‐seq creatively introduced the concept of “compressed sensing” [167]. Instead of confining each cell to a single perturbation, this approach involved subjecting each cell to a random combination of multiple perturbations or encapsulating multiple cells bearing distinct perturbations within a single droplet. By employing algorithms like FR‐Perturb to deconvolve individual perturbation effects from mixed expression signals, this method achieved an order‐of‐magnitude reduction in screening costs while maintaining accuracy comparable to traditional approaches. The first application of Perturb‐seq in live animals came in 2020, when Zhang et al. developed in vivo Perturb‐seq, successfully extending this technology to the living organism in a pivotal leap from in vitro cell lines to complex biological systems [168]. The researchers performed in utero lentiviral injections in mice during embryonic development to deliver a gRNA library targeting 35 genes associated with autism spectrum disorder (ASD)/neurodevelopmental delay, followed by single‐cell sequencing of the postnatal mouse brain. The analysis revealed that perturbations of these risk genes elicited cell‐type‐specific transcriptomic alterations in distinct neuronal populations, uncovering their critical roles in cell fate decisions and identifying several gene expression modules conserved in human autism patients. Subsequently, several in vivo CRISPR screening platforms have been developed, including CrAAVe‐seq, CRISPR‐StAR, and in vivo barcoded CRISPR–Cas9 screening, which expanded the application of pooled genetic screening to complex tissue environments [90, 131, 169, 170, 171].

#### 2.4.4 High Content Read‐Out Beyond Transcriptomics

In recent years, Perturb‐seq has progressively expanded from transcriptome‐only readouts toward chromatin and spatial transcriptomic analysis to acquire more information from functional dimensions such as protein levels, posttranslational modifications, metabolic states, and spatial localization.

In 2019, Perturb‐ATAC pioneered the integration of CRISPR screening with single‐cell chromatin accessibility profiling, enabling simultaneous detection of genetic perturbations and chromatin accessibility states at single‐cell resolution, thereby providing a new tool for linking gene regulation with epigenetic states [172]. Aiming for higher resolution, researchers subsequently developed TDAC‐seq, which introduces mutations in accessible chromatin regions using the double‐stranded DNA cytidine deaminase *DddA11*. By combining targeted PCR amplification with nanopore long‐read sequencing, this technique first achieves the simultaneous readout of CRISPR editing and chromatin accessibility information on individual chromatin fibers. In a high‐throughput CRISPR scan of the *GFI1B* enhancer in hematopoietic stem/progenitor cells, TDAC‐seq successfully identified transcription factor motifs, including *GATA*, *AP‐1* and *SPI1* that regulate enhancer accessibility [173]. Whereas Perturb‐ATAC and TDAC‐seq capture static snapshots of chromatin states at single‐cell and single‐molecule resolution, respectively, the CRISPR PRO‐LiveFISH technology developed by Liu et al. in 2025 extends this investigative scope into the dynamic dimension. Built upon an expanded genetic alphabet and a Cas9‐based multicolor imaging platform in living cells, this technique enables real‐time visualization of nonrepetitive DNA sequences [174]. This breakthrough allows researchers to track the dynamic behavior of specific genomic loci in live cells and investigate real‐time processes of chromatin motion and enhancer–promoter interactions. Through CRISPR PRO‐LiveFISH, the authors revealed an association between active epigenetic states and reduced chromatin mobility. Demonstrating that enhancer–promoter interactions can persist during dynamic motion, they further established that *BRD4* is critical for maintaining 3D interactions between the *MYC* oncogene and its super‐enhancer. These findings underscore the immense potential of live‐cell imaging‐based screening for studying dynamic processes.

Spatial transcriptomics read‐out enables researchers to dissect the spatial heterogeneity of gene expression, intercellular communication networks, and the functional architecture of tissue microenvironments while preserving the native in situ positional information of cells. In 2018, the protein‐level barcoding system Pro‐Code achieved high‐dimensional protein detection at single‐cell resolution. This system uses a limited set of antibody‐detectable epitopes arranged in specific multiples to form a large number of unique barcodes. By pairing each Pro‐Code with a different CRISPR, researchers successfully identified *Psmb8* and *Rtp4* as key genes regulating antigen‐specific T‐cell killing and uncovered *Socs1* as a negative regulator of PD‐L1, demonstrating the unique value of protein‐dimension screening [11]. The core advantage of Pro‐Code lies in its compatibility with mass cytometry (CyTOF), enabling simultaneous measurement of dozens of protein markers at single‐cell resolution and extending CRISPR screening readouts from the transcriptome to the proteome.

Capitalizing on this system, Perturb‐map technology in 2022 achieved the first integration of spatial transcriptomics with in vivo CRISPR screening. By knocking out 35 genes in a mouse model of lung cancer, the researchers found that *Tgfbr2* knockout promotes TME remodeling and immune exclusion, revealing the critical importance of spatial tissue context in interpreting gene function [175]. With these foundations in place, a wave of spatial multimodal screening technologies has recently emerged. Technologies such as Perturb‐FISH and CRISPRmap combine in situ sequencing with multiplexed imaging to map perturbation‐phenotype landscapes within intact tissues and cells [176, 177]. CLIM–TIME integrates in vivo CRISPR screening with laser capture microdissection (LCM) and spatial transcriptomics. Through systematic knockout screening of 391 tumor suppressor genes, this approach achieved the simultaneous dissection of gene perturbations, spatial localization, and microenvironmental cellular composition within a single experiment. With this platform, the authors identified seven metastatic niche subtypes and discovered that deletion of the Hippo pathway gene *Nf2* establishes an immunosuppressive microenvironment enriched for myeloid cells but depleted of T cells, mediated through *LOXL2*‐driven collagen crosslinking. Targeting *LOXL2* effectively remodels this microenvironment and substantially enhances T‐cell therapy efficacy. The core contribution of CLIM–TIME lies in marrying the spatial resolution of microdissection with the systematic power of functional screening, enabling researchers to dissect how genetic perturbations shape local microenvironments in situ and directly translate these findings into combinatorial therapeutic strategies and biomarkers [178].

Whereas conventional screening methods are limited to readouts of single phenotypes or population‐averaged signals, the deep integration of CRISPR technology with high‐dimensional readouts, particularly spatial transcriptomics, has elevated functional genomics screening from coarse‐grained resolution of “which genes regulate a phenotype” to a systems‐level understanding of “how genetic perturbations remodel cellular states and molecular networks in situ within the native tissue architecture.”

### 2.5 The Comparison Between RNAi and CRISPR Screening

RNAi dates back to 1998, when Fire and Mello first demonstrated in C. elegans that double‐stranded RNA could sequence‐specifically silence gene expression [179]. In 2001, Tuschl et al. further showed that chemically synthesized 21–23 nucleotide siRNAs could achieve efficient gene knockdown in mammalian cells, ushering in the era of RNAi as a tool for functional genomics [180]. The mechanism of RNAi relies on the endogenous RNA‐induced silencing complex: upon siRNA loading, the guide strand directs the complex to complementary mRNAs, leading to their cleavage or translational repression at the posttranscriptional level [181]. An RNAi screen is essentially a forward genetics screen using a reverse genetics technique, bringing two major advantages over classical genetic screens: the sequences of all identified genes are immediately known, and lethal mutations are easier to identify because it is not necessary to recover mutants. These features enabled RNAi to systematically interrogate gene function on a genome‐wide scale with unprecedented efficiency and throughput [182]. Leveraging its rapid and accessible nature, RNAi screening catalyzed numerous important discoveries over the following decade, particularly in pathway dissection and cancer biology. However, as applications expanded, the inherent limitations of RNAi technology became increasingly apparent, distinguishing it from CRISPR‐based approaches (Table 3).

**TABLE 3:** Comparison of RNAi screening and CRISPR screening.

	RNAi screening [179, 180, 181].	CRISPR screening [27, 28, 36, 38, 44, 183, 184].		
		CRISPR knockout	CRISPRi (dCas9–KRAB)	CRISPRa (dCas9–VPR)
Mechanism	Posttranscriptional mRNA degradation, translational repression	NHEJ‐mediated frameshift/stop codon	Transcriptional repression via KRAB domain (steric hindrance + H3K9me3)	Transcriptional activation via VP64/p65/HSF1 recruitment
Result	Reversible knockdown	Permanent knockout	Reversible knockdown	Reversible overexpression
Transgenes	si/shRNA	Cas9 nuclease + gRNA	dCas9–KRAB + gRNA	dCas9–VPR + gRNA
Transcript variant handling	All variants via conserved region	All variants via conserved region	Only variants from the same TSS	Only variants from same TSS (promoter)
Typical readout	Low‐dimension, population‐averaged	Perturb‐seq, ATAC, spatial transcriptomics	Same as CRISPR KO	Same as CRISPR KO
Advantage	No DNA damage; suitable for redundant genes, dose‐sensitive processes	Permanent loss‐of‐function, high reagent consistency, integration with single‐cell multiomics	Reversible, no DSB, high specificity, ideal for large scale regulatory network mapping	Endogenous activation preserves native regulation, overcome cDNA library limitations
Limitation	Incomplete knockdown (false negatives); seed‐mediated off‐targets (high false positives)	Potential severe toxicity or lethality, small in‐frame indels may retain residual function; PAM restriction	Incomplete knockdown, requires precise TSS annotation, ineffective in some species/cells	Uneven activation efficiency across genes, off‐target transcriptional risks

The fundamental distinction lies in the level at which each technology intervenes in genetic information. RNAi operates posttranscriptionally, where siRNAs guide the RISC complex to induce degradation or translational inhibition of target mRNAs. As a result, siRNAs almost never fully deplete the target mRNA, and usually several different siRNAs must be screened before an effective siRNA is identified. Transient RNAi in rapidly dividing tissue culture cells typically lasts 3–5 days. However, even when applied to slowly dividing cells, an effective siRNA may still have difficulty depleting a stable protein [181]. CRISPR–Cas9, by contrast, enables permanent and irreversible genetic perturbations. The Cas9 nuclease, guided by a sgRNA, introduces DSBs at specific genomic loci. Repair via NHEJ generates frameshift mutations or premature stop codons, resulting in complete gene knockout [183, 184]. Notably, the CRISPR platform has since expanded beyond knockout to include inducible transcriptional repression systems such as CRISPRi (dCas9–KRAB), offering alternative strategies for essential gene studies [27].

Off‐target effects represent the predominant source of false positives in functional genomics screens, yet RNAi and CRISPR exhibit qualitatively distinct off‐target profiles. RNAi off‐targeting arises from mechanistic overlap with the endogenous miRNA pathway. Upon RISC loading, the “seed region” (nucleotides 2–8 at the 5′ end of the guide strand) requires only partial complementarity to 3′ untranslated regions of unintended transcripts to induce miRNA‐like translational repression or degradation. This seed‐mediated off‐targeting is mechanistically intrinsic to RNAi and cannot be fully circumvented by sequence optimization, leading to substantial multitargeting, persistently high false‐positive rates, and an elevated validation burden [185]. In contrast, off‐target effects in CRISPR systems are predictable and avoidable. Cas9 target recognition requires near‐continuous complementarity over approximately 20 nucleotides between the sgRNA and genomic DNA, in conjunction with the protospacer adjacent motif (PAM). Off‐target cleavage thus necessitates extensive sequence homology, enabling systematic prediction and avoidance using computational tools such as CFD scoring [23, 186]. Moreover, dCas9‐based platforms such as CRISPRi/a operate without inducing DNA DSBs, further mitigating off‐target risks [187].

RNAi readouts remained limited to low‐dimensional, population‐averaged measurements. Critically, due to the transient nature of RNAi perturbations and reliance on endogenous RISC machinery, precise linkage between perturbation identity and scRNA‐seq profiles was unattainable [188, 189]. CRISPR technologies have transformed readout dimensionality across two key fronts. On one hand, the deep integration of CRISPR screening with scRNA‐seq, exemplified by Perturb‐seq and CROP‐seq, enables whole‐transcriptome information at single‐cell resolution. On the other hand, CRISPRa/i platforms offer flexible coupling with diverse readout modalities, enabling dynamic monitoring of signaling pathways via fluorescent reporters, enrichment of specific cell states through surface marker‐based flow sorting, and genome‐wide profiling of epigenetic remodeling when integrated with ATAC‐seq.

## 3 Biological and Biomedical Applications

Armed with the advanced methodologies described above, high‐content CRISPR screening is driving paradigm shifts across multiple biomedical fields. This section comprehensively reviews transformative applications of CRISPR screening. We first explore its impact on oncology specifically in mapping tumorigenesis, metastasis, and drug resistance and its role in optimizing immunotherapies such as CAR T‐Cell (CAR‐T) and CAR‐NK cell therapies. Subsequently, we highlight key discoveries facilitated by CRISPR screening in developmental biology, neurobiology, and infectious diseases, culminating in an evaluation of how these screen‐identified targets are currently progressing through clinical trials.

### 3.1 Cancer Biology and Drug Resistance

Molecular mechanisms driving tumorigenesis, metastasis, immune evasion, and therapeutic resistance remain a central challenge. High‐content CRISPR screening has emerged as a pivotal unbiased functional genomics platform to systematically interrogate these complex processes. In this section, we summarize the pivotal applications of CRISPR screening across these four core areas of cancer biology and drug resistance.

#### 3.1.1 Identification of Genes Associated With Tumorigenesis

According to the evolutionary theory of cancer, driver mutations arise stochastically in phenotypically normal tissues prior to malignant transformation, promoting clonal expansion. Nevertheless, the mechanisms underlying why only a subset of these mutated clones progresses to full malignancy remain poorly understood. Systematic identification of protumorigenic genes via CRISPR screen provides novel insights into therapeutic strategies targeting early‐stage tumor development. Shalem et al. pioneered the application of the GeCKOv2 library to systematically identify genes critical for cellular viability in human melanoma and PSCs, revealing essential factors such as *NF2*, *CUL3*, and *TADA2B* [19]. Building on this methodology, Behan et al. established a comprehensive data‐driven framework for therapeutic target prioritization based on 324 cancer cell lines representing 30 cancer types, identifying candidate therapeutics targets including *Cdkn2a*, *USP15*, and *SCAF1* [190]. Tumor heterogeneity and host‐versus‐graft reactions impede in vivo CRISPR screens in complex models such as 3D cell cultures, organoids, cell line‐derived xenografts, and patient‐derived xenografts (PDX). CRISPR‐StAR overcomes these limitations using Cre‐regulated stochastic sgRNA activation with integrated single‐cell barcoding. By segregating active/inactive sgRNA states during clonal expansion, it establishes intrinsic single‐cell controls to decouple biological noise from technical variability. Applying CRISPR‐StAR to genome‐wide screens in murine and human melanoma models, researchers uncovered aerobic metabolism as a critical requirement for in vivo tumor growth, evidenced by significant enrichment of mitochondrial inner membrane‐related genes—a finding obscured in vitro by nonphysiological culture conditions. Additionally, the screen identified tumor suppressors such as *Stk40* and *Zbtb10*, which are required for tumor growth specifically in vivo [191]. Pooled CRISPR screens excel in assessing gene essentiality in tumor proliferation and progression; however, simple readouts averaged over all tissue cell types are their limitation [192]. Integrating CRISPR screening with single‐cell sequencing and spatial functional genomics now enables functional genomic analysis at tissue‐scale resolution. Pioneering this approach, Brown's team developed Perturb‐map, an in vivo spatial functional genomics platform that preserves tissue architecture while interrogating tumors at single‐cell resolution by employing protein barcodes and multiplexed imaging to spatially resolve CRISPR perturbations. By applying a nuclear Pro‐Code/CRISPR LV library targeting 35 cytokine signaling and immune interaction‐related genes to lung cancer cells, they demonstrated that loss of *Tgfbr2* and *Socs1* induces distinct lesion phenotypes in Kra^G12D^p53^−/−^ tumors while cooperatively promoting tumor growth [175]. In vivo single‐cell CRISPR screens systematically profile the 150 most frequently mutated driver genes in squamous cell carcinomas, including human head and neck squamous cell carcinoma (HNSCC) and skin squamous cell carcinoma. Researchers delivered a lentiviral sgRNA library into the Cas9‐expressing amniotic cavity of E9.5 mouse embryos to generate LOF mutations. Epidermal tissues were harvested, enriched for mCherry^+^ infected cells, and subjected to scRNA‐seq at postnatal Days 4 (P4) and 60 (P60). This approach revealed that clonal expansion within phenotypically normal skin tissues correlates with TNF signaling activation, and the transition to autocrine TNF production marks a critical checkpoint in early tumorigenesis [192]. Wang et al. integrated in vivo CRISPR screening with laser‐captured microdissection to achieve spatial mapping of the metastatic TME. Through a systematic screen of 391 tumor suppressor genes, they discovered that loss of Hippo pathway TSGs (e.g., Nf2) drives the formation of an immunosuppressive, myeloid‐rich yet T‐cell‐excluded niche by upregulating *LOXL2*‐mediated collagen crosslinking [178]. He et al. employed CRISPR screening in a mouse gastric organoid model to reveal the central roles of the Pten/Akt and TGF‐β signaling pathways in gastric tumorigenesis. The study further established that Helicobacter pylori promote tumor growth by recruiting SiglecF^+^ neutrophils [141].

#### 3.1.2 Identification of Genes Associated With Tumor Metastasis

Tumor metastasis operates as a multistep cascade requiring coregulation by intrinsic oncogenic lesions and extrinsic spatiotemporal interactions with the TME [193].

Despite therapeutic advances, metastatic disease remains the predominant cause of cancer‐related mortality [194], necessitating deeper mechanistic insights and actionable therapeutic targets. To systematically interrogate genetic drivers of metastasis, Chen's team engineered massively parallel CRISPR–Cpf1/Cas12a crRNA array profiling, a dual‐gene knockout platform targeting 325 metastasis‐associated gene pairs via about 774,000 unique barcode tags. Following lentiviral transduction of non‐small cell lung cancer (NSCLC) cells and subcutaneous engraftment into immunodeficient (nu/nu) mice, the pioneering in vivo dual‐gene screen identified synergistic prometastatic pairs, most notably *Nf2_Trim72* and *Nf2_Chd1*, which orchestrate lung metastasis [195]. Parallel studies addressing metastatic inefficiency focused on CTCs, which disseminate from primary tumors into circulation and ultimately initiate metastases. Patient‐derived CTCs were transduced with sgRNA and intravenously injected into mice, and low‐burden lung metastases harvested after 2 months underwent sgRNA enrichment analysis. High‐scoring hits—including ribosomal biosynthesis genes (*RPL15*, *RPL35*, *RPL13*)—revealed that metastatic fitness in CTCs critically depends on augmented protein synthesis [196]. An in vivo CRISPRi screening identified a gene named Mitochondrial Fission Process Protein 1 (*MTFP1*) as a key driver of liver metastasis in pancreatic ductal adenocarcinoma (PDAC). The study revealed that *MTFP1* promotes the colonization and growth of hepatic metastases by remodeling the mitochondrial network and directly enhancing ATP synthase activity. This reprograms tumor cell metabolism toward glutamine utilization and oxidative phosphorylation (OXPHOS), while simultaneously competing with CD8^+^ T cells in the TME for glutamine, resulting in immunosuppression [197]. Collectively, these CRISPR‐enabled discoveries not only decipher cooperative gene pairs driving metastatic progression but also illuminate ribosomopathy and mitochondrial disorder as unappreciated axis of dissemination fitness, thereby nominating cotargeting strategies against metastatic resilience as a frontier for therapeutic development.

Conventional CRISPR screening approaches predominantly interrogate gene perturbations in cancer cells. In contrast, Moor's team at ETH Zurich developed an innovative strategy by introducing gRNAs into nonproliferative hepatocytes, ensuring stable library distribution throughout the experimental period. Their methodology employed the Cre–loxP system to achieve liver‐specific expression of dCas9–SPH and used hydrodynamic tail vein injection to deliver plasmids containing sgRNAs and Sleeping Beauty transposase (*SB100X*). This approach enabled liver‐specific gene activation and created a “mosaic liver” model featuring multiple perturbed microenvironments. Following intrasplenic injection of mCherry‐labeled tumor cells, comparative analysis of sgRNA enrichment patterns in distal versus proximal hepatocytes surrounding metastatic foci identified liver‐derived chemokines and axon guidance signals as key regulators of metastatic seeding. Notably, the Plexin B2–semaphorin–KLF4 signaling axis emerged as a central metastatic determinant [198]. Innovatively, this research focuses on determinants in the colonization environment instead of metastatic entities, and future researchers can explore potential targets in other organs using a similar approach. The team led by Andrew P. Feinberg conducted a CRISPR‐based genome‐wide functional screen using paired PDX models, systematically mapping for the first time the differences in gene essentiality between primary human pancreatic cancer and distant metastatic lesions. The study revealed that the transcription factor *KLF5* is a specific dependency for the proliferation of metastatic pancreatic cancer cells. Knockdown of *KLF5* reversed the widespread loss of heterochromatin observed in metastases and significantly suppressed metastatic cell growth and migration. More importantly, the research uncovered an epigenetic regulatory cascade: KLF5 → NCAPD2/MTHFD1 → chromatin modification → metastasis‐associated genes (*TGFBR2*/*VIM*/*EMP1*/*ITGB1*) [199].

#### 3.1.3 Identification of Genes Associated With Tumor Immune Evasion

Cancer cells evade immune surveillance through antigen downregulation, immunosuppressive cytokine secretion, and microenvironmental remodeling, thereby facilitating tumor proliferation and metastatic dissemination [200, 201]. CRISPR‐based screening has systematically identified genes orchestrating immune evasion, offering critical insights into therapeutic vulnerabilities.

For example, a genome‐wide CRISPRko screening across six cancer models—including renal carcinoma (Renca‐HA), melanoma (B16‐Ova), breast cancer (4T1‐HA, EMT6‐HA), and colorectal cancer (CT26‐HA, MC38‐Ova)—revealed 182 “Core Cancer Intrinsic Immune Evasion Genes” converging on pathways governing IFNγ signaling, antigen presentation, NF‐κB signaling, autophagy, mTOR signaling, and mitochondrial metabolism. Notably, *Fitm2* was revealed as a novel mediator of immune evasion by sustaining tumor cell survival under IFNγ exposure [202]. Complementing these findings, Liu's team employed an in vivo CRISPRko screening with the MusCK library in triple‐negative breast cancer (TNBC) models, demonstrating that Cop1‐deficient TNBC cells exhibit heightened susceptibility to immune clearance through Cop1's mechanistic role in degrading C/ebpδ via Cop1–Trib2 interaction, thereby suppressing macrophage‐attracting chemokine secretion and tumor infiltration [68]. Integrated analysis of transcriptomic, proteomic, and in vivo CRISPR screening reveals that the endoplasmic reticulum ribosomal‐binding protein 1 (*RRBP1*) is highly expressed in bladder cancer. *RRBP1* promotes tumor proliferation and metastasis while suppressing the function of CD8^+^ T cells in the TME [203]. Song et al. conducted an in vivo CRISPR screening in TNBC using a knockout library targeting 581 cell surface protein genes. The study revealed that tumor cells utilize the R1 domain of tumor‐intrinsic *ITGB1* to reprogram tumor‐associated macrophages (TAMs) into an M2‐like phenotype that promotes tumor growth and suppresses immunity, thereby fostering an immunosuppressive microenvironment conducive to tumor progression [204]. Fu et al. performed a CRISPR screening using a sgRNA library targeting 936 epigenetic regulators and discovered that ubiquitin C‐terminal hydrolase 5 (*UCHL5*) positively regulates the expression of extracellular matrix‐related genes by stabilizing its interacting protein *NFRKB*. This promotes epithelial–mesenchymal transition (EMT) and stromal fibrosis, thereby impairing immune cell infiltration into the TME [205]. In summary, these studies demonstrate that diverse tumor‐intrinsic genetic alterations promote immune evasion primarily by altering the TME to restrict protective immune cell infiltration.

Among the numerous immune evasion strategies employed by tumor cells, the upregulation of programmed PD‐L1 stands as one of the most representative key mechanisms. Its expression undergoes intricate, multitiered regulation spanning genetic, epigenetic, transcriptional, and posttranslational modifications. Unravelling this regulatory network is central to overcoming resistance to immune checkpoint inhibitors (ICBs). CRISPR screening affords comprehensive delineation of critical genes and signaling pathways governing the expression and functional modulation of PD‐1/PD‐L1, spanning epigenetic reprogramming, transcriptional regulation, and posttranslational modifications. Chromatin remodeling at the PD‐L1 locus (chromosome 9p24.1) directly regulates its transcriptional activity. Through a FACS‐based CRISPR screen with dual PD‐L1/IRF3 staining, SMARCAL1 was identified from a 609‐gene library as a potent regulator of PD‐L1. Mechanistically, SMARCAL1 enhances PD‐L1 expression by simultaneously limiting endogenous DNA damage and maintaining chromatin accessibility [206]. Many transcriptional regulators are postulated to modulate PD‐L1 expression. Two melanoma‐focused CRISPR screening studies have elucidated the transcription factor modifications in PD‐L1 regulation. By employing a library targeting 96 deubiquitinating enzymes, *ATXN3* was demonstrated as a PD‐L1 upregulator via the protection of transcription factors (IRF1, STAT3, HIF‐2α) and deubiquitination of the AP‐1 transcription factor JunB [207]. In parallel, a CRISPRi screening targeting 3323 proteostasis‐related genes revealed that TRAF6 mediates lysine‐63 (K63) polyubiquitination to stabilize the formation of the YAP1/TFCP2 transcriptional complex, thereby enhancing PD‐L1 transcription [208]. Meanwhile, a genome‐wide screening in NSCLC highlighted that N‐terminal acetylation of LIP facilitates its nuclear entry and subsequent interaction with *BHLHE22*, forming a corepressor complex at the PD‐L1 promoter that effectively downregulates PD‐L1 expression [209].

Beyond intrinsic transcriptional control, PD‐L1 expression is dynamically driven by extrinsic microenvironmental cues, primarily IFN‐γ signaling activated during tumor–immune interactions. An innovative “two cell type”‐CRISPR assay, employing human T cells as effectors and melanoma cells as targets, revealed trans‐cellular regulatory mechanisms in tumor–immune interactions. *APLNR* deficiency in melanoma cells was unraveled to impair CD8^+^ T cell‐mediated cytotoxicity by disrupting JAK1‐dependent IFN‐γ signaling, ultimately diminishing the efficacy of both adoptive cell therapy and immune checkpoint blockade [210]. Complementary in vitro CRISPR screening in melanoma models identified the tyrosine phosphatase *PTPN2* as an inhibitor of IFN‐γ signaling through its dephosphorylation of STAT1 and JAK1. To translate preclinical findings, subsequent clinical trials for solid tumors were conducted. The compound ABBV‐CLS‐484 is a first‐in‐class, orally bioavailable inhibitor targeting both *PTPN2* and *PTPN1*, which improves the cytotoxic capacity and persistence of antitumor immune cells through multiple immune signaling pathways, including IFN‐γ response, TCR signaling, and IL‐2/7/15 pathways [211, 212].

Genome‐wide screening across multiple cancer cell lines revealed discrete mechanisms of posttranscriptional regulation of PD‐L1/PD‐1, spanning mRNA stability, protein stability, and subcellular localization. With respect to mRNA stability, an in vivo CRISPR LOF screening in TNBC models uncovered that intracellular CD28 directly associates with CD274 mRNA and recruits spliceosomal factor *SNRPB2* to stabilize CD274 mRNA in nucleus, elevating PD‐L1 expression and immune escape [213]. As for protein stability, the genome‐wide CRISPR screening in GC cells demonstrated that *TRIM28* directly binds to PD‐L1 and prevents its proteasome‐dependent degradation via suppression of PD‐L1 ubiquitination and induction of PD‐L1 SUMOylation. Coordinately, *TRIM28* facilitates K63 polyubiquitination of TBK1, with downstream effects of activating TBK1–IRF1 and TBK1–mTOR pathway. This combined action amplifies PD‐L1 abundance [214]. Furthermore, two genome‐wide screens in pancreatic cancer cells and melanoma cells separately confirmed that CMTM6 could colocalize with PD‐L1 both at the plasma membrane and recycling endosomes, shielding PD‐L1 from lysosomal degradation. Notably, Izar's team integrated scRNA‐seq to delineate that the intact CD58‐CD2 axis on tumors directly impedes T cell infiltration, coupled with a parallel mechanism where CD58 competition between CD58 and PD‐L1 for *CMTM6* binding destabilizes PD‐L1 [215, 216]. Finally, extending beyond classical proteostasis, a dual approach combining a high‐content metabolic compound library with a genome‐wide CRISPR screening linked PD‐L1‐mediated resistance to metabolic reprogramming. This study identified dihydroorotate dehydrogenase as a driver of anti‐PD‐1 resistance by regulating tumor cell macropinocytosis, establishing a direct functional link between nutrient acquisition pathways and immune evasion [217].

#### 3.1.4 Dissecting Drug Resistance Mechanisms via CRISPR Screening

Elucidating the intricate molecular networks of drug resistance is critical for designing durable cancer therapies. CRISPR screening is uniquely equipped to decode these adaptive responses across diverse cancer types. In the realm of targeted therapies, CRISPR screens have effectively unraveled resistance mechanisms lagging behind small‐molecule inhibitors and tyrosine kinase inhibitors (TKIs). For instance, prolonged CDK4/6 inhibitor treatment in hepatocellular carcinoma (HCC) triggers BAP1‐dependent chromatin remodeling, driving an epigenetic state that activates WNT and EMT signaling to enhance cell survival [62]. In KRAS‐mutant PDAC, a two‐step genome‐wide CRISPRko screening employing the Brunello library followed by a focused secondary library systematically revealed that acquired resistance to combined SHP2 and ERK inhibition is primarily driven by *PTEN* loss, which activates the PI3K–AKT–mTOR pathway and sustains activation of the downstream transcription factor JUN (cJun) [218], while a parallel screening centered on KRAS inhibitors uncovered a complex resistance network involving the EGFR–RAS–ERK, Hippo–YAP, and CK2–LEF1–β‐catenin axes, and further identified novel synergistic targets including CK2 and PI3Kγ [219]. Furthermore, to address first‐line TKI resistance in advanced HCC and NSCLC, CRISPR–Cas9 synthetic lethality and proteomic‐coupled screens identified ERBB3 (HER3) as a key factor limiting second‐line regorafenib efficacy [220], TGF‐β receptor‐binding protein 1 (*TGFBRAP1*), which thwarts Smurf1/2‐mediated ubiquitination and proteasomal degradation of *TGFBR1* through competitive binding, thereby amplifying downstream TGF‐β signaling to sustain liver cancer stemness and TKI resistance [221]. Similarly, in advanced prostate cancer, specialized high‐content and CRISPRi screens coupled with FACS identified *ZMYND8* and *PTGES3* as crucial regulators of therapy‐induced neuroendocrine differentiation and androgen receptor modulation, respectively, leading to the development of synergistic small‐molecule inhibitors [151, 222].

In addition to targeted drug failure, CRISPR screening has systematically interrogated the genetic determinants of classical chemoresistance, which remains a primary cause of treatment failure in bone and gynecological malignancies. In osteosarcoma, a targeted CRISPRko screening focusing on the human kinome identified the DNA‐dependent protein kinase catalytic subunit (PRKDC) as a central determinant of doxorubicin sensitivity, providing a novel therapeutic target to overcome clinical chemoresistance [223]. Complementing this, a combined transcriptomic and kinome‐wide high‐content screen highlighted the protein kinase *PKMYT1* as a negative regulator of cisplatin sensitivity, demonstrating that the selective *PKMYT1* inhibitor RP6306 exhibits a marked synergistic antitumor effect when combined with cisplatin [224]. Moreover, utilizing CRISPR screening paired with FACS flow cytometry, researchers delineated how the protein PRMT5 drives acquired resistance to platinum‐based chemotherapy in ovarian cancer cells [225]. These functional genomics findings offer novel strategies to restore chemosensitivity via rational combination regimens.

Finally, CRISPR screens have been extensively deployed to systematically decode the multifactorial resistance networks driving therapeutic refractoriness in hematologic malignancies. At the genomic and DNA damage response (DDR) level, a genome‐wide screen in hematopoietic stem and progenitor cells demonstrated that loss of the checkpoint kinase *CHEK2* confers profound resistance to both conventional genotoxic chemotherapies (cisplatin and melphalan) and DNA‐hypomethylating agents [226]. Beyond genomic maintenance, disrupting target protein stability and turnover represents another pivotal bypass mechanism. In CML models, a genome‐wide CRISPR screen identified that loss of the *KCTD5* gene drives TKI resistance by impairing the ubiquitination and proteasomal degradation of the oncogenic BCR::ABL1 fusion protein [227]. Concurrently, bypassing transcriptional control via aberrant translational fidelity has emerged as a novel resistance axis. Utilizing a dual‐dimensional CRISPR screen that couples nascent protein labeling with cell proliferative capacity, researchers identified *PABPC1* as a critical translation regulator in the blast crisis phase of CML; mechanistically, PABPC1 selectively accelerates the translation of proleukemic mRNAs through a phase‐separation mechanism to drive disease progression and TKI resistance [228]. These studies highlight the pivotal role of CRISPR screening in systematically elucidating cancer resistance mechanisms and guiding the development of novel therapeutic strategies.

### 3.2 Immunology and Immunotherapy

#### 3.2.1 Regulators of T Cell Differentiation

CRISPR screening can be applied to identify key genes regulating T cell activation, proliferation, differentiation, toxic effects, and exhaustion, thereby offering novel therapeutic targets for optimizing cancer immunotherapy.

*FOXP3*‐expressing regulatory T (T_reg_) cells, balancing the immune responses against self‐antigens and tumor, are correlated with poor prognosis when extensively infiltrating tumor tissues. FOXP3 emerges as a key therapeutic target, rebalancing the dynamic equilibrium between effector T (T_EFF_) cell activation and immunosuppressive population control. Pooled and arrayed Cas9 RNP screens fused with 54,424 single‐cell transcriptomic profiling of T_reg_ cells under combinatorial genetic perturbations and cytokine milieus, unmasking three regulatory axes: *FOXP3* and *PRDM1* driven nonoverlapping gene networks alongside context‐dependent transcriptional hierarchies governed by FOXO1–IRF4 coregulated circuitry [229]. To deconvolve the Foxp3 regulatory network, a targeted LOF CRISPR screening of 489 nuclear regulators was conducted. This screen revealed that Usp22 transcriptionally enforces *FOXP3* through histone deubiquitination while posttranscriptionally stabilizing its expression; a parallel mechanistic study further identified *RNF20* as a repressive ubiquitination mark [230]. A genome‐wide CRISPR screen integrating intracellular protein detection in human primary T cells systematically identified the RBPJ–NCOR complex as a novel regulator of the key transcription factor *FOXP3*. This led to the development of a genetic editing strategy that stably enhances the function of induced T_reg_ cells [59]. Similarly, a genome‐wide CRISPR LOF screen was conducted in murine primary T_reg_ cells to decode *FOXP3* regulatory architecture, with convergent enrichment detected in SWI/SNF nucleosome‐remodeling complex subunits and SAGA histone acetyltransferase modules. Among the three SWI/SNF‐related complexes, the Brd9‐containing noncanonical BAF complex promoted *FOXP3* expression, whereas the polybromo‐associated BAF complex was repressive [231]. Rathmell et al. demonstrated that *MTHFD2* deficiency, impeding mitochondrial one‐carbon metabolism, depletes purine pools, accumulates purine biosynthetic intermediates, and attenuates mTORC1 signaling [232].

In contrast to immunosuppressive T_reg_ cells, memory T cells (T_MEM_) rapidly expand and differentiate into T_EFF_ upon antigen re‐exposure, conferring long‐term immune protection. A CRISPR screening was deployed to interrogate negative regulators of T_MEM_ generation in vivo, revealing that during the first division of activated CD8^+^ T cells, cBAF and c‐Myc frequently coassort asymmetrically to the two daughter cells—those inheriting elevated c‐Myc/cBAF levels commit to a T_EFF_ cell fate trajectory, whereas counterparts with diminished c‐Myc/cBAF are predisposed to T_MEM_ differentiation [233]. As it pertains to nutrient‐immune crosstalk, an in vivo pooled CRISPR screen was established to dissect metabolic determinants of T_EFF_/T_MEM_ lineage bifurcation, identifying the amino acid transporters *SLC7A1* and *SLC38A2* as critical rheostats orchestrating T_MEM_ response kinetics through mTORC1 signaling‐mediated metabolic circuit integration. *SLC7A1* and *SLC38A2* attenuate T_MEM_ differentiation, partially via mTORC1 signaling suppression. Amino acid deprivation or deficiencies in lysosomal signaling nodes (Flcn, Ragulator, Rag GTPases) induce T_feb_ to drive T_RM_ formation. Mechanistically, the Flcn–T_feb_ axis restrained retinoic acid‐induced CCR9 expression for migration and TGF‐β‐mediated programming for lineage differentiation [234]. Marson et al. developed a CRISPRa and CRISPRi discovery platform in primary human T cells and performed genome‐wide screens for functional regulators of cytokine production in response to stimulation. Their study not only demonstrated tumor necrosis superfamily receptor including 4‐1BB, CD27, CD40, and OX40 as activators of T cell cytokine production but also uncovered that genes like *VAV1* and *FOXQ1* dictate differentiation trajectories through polarized regulation—preferentially increasing Type 1 signature cytokines while dampening Type 2 cytokines [235]. Building upon prior screening, a targeted CRISPRa array screening to investigate stemness maintenance in CD8^+^ T cells was engineered, revealing LMO4 transcriptional activation expands stem‐like memory CD8^+^ T cell reservoirs through IL‐21–STAT3 axis reprogramming, thereby improving antitumor efficacy [236].

#### 3.2.2 Regulators of T Cell Activation

In 2020, Phillips et al. conducted genome‐wide CRISPRko screening using the GeCKOv.2 library, elucidating that the T cell clone MC.7.G5 exhibited potent cytotoxicity via TCR recognition of cancer‐specific metabolic markers presented by MR1 [237]. Through a synergistic approach that combined a genome‐wide CRISPR screening with protein–protein interaction network mapping, Chi et al. identified a functionally critical SEC31A–SEC13 interface within the architecture of the nutrient‐sensing GATOR2 complex and concurrently demonstrated that the SWI/SNF complex enhances mTORC1 activation by repressing the expression of the amino acid sensor CASTOR1 [238]. In 2023, Marson et al. employed a genome‐wide screen in γδ T cells to mechanistically pinpoint *BTN3A* as the central orchestrator of Vγ9Vδ2 T cell effector functions; AMPK agonism augments cytotoxicity via BTN2A1–BTN3A axis modulation [239]. Concurrently, comprehensive CRISPRa‐driven interrogation of the transmembrane proteome was executed, culminating in the definitive identification of *MUC‐21* as an immunosuppressive gatekeeper that impairs antigen recognition and suppresses T cell activation [65]. The PerturbSEQ methodology, uniting CRISPR‐mediated gene perturbation with scRNA‐seq, was further developed into Perturb‐CITE‐seq. Deploying this platform across 28 IL‐2Rα regulatory nodes, Marson et al. revealed MED12 as a central calibrator of T cell activation dynamics. Through state‐specific epigenetic reprogramming, MED12 orchestrates the critical transition from quiescence to full functional activation [126].

#### 3.2.3 Regulators of T Cell Proliferation

In 2016, Marson et al. established RNP complexes for genome editing in primary CD4^+^ T cells. Building upon this method, they achieved a genome‐wide LOF screening in primary human T cells by merging lentiviral sgRNA delivery with Cas9 protein electroporation, termed SLICE (single gRNA lentiviral infection with Cas9 protein electroporation). *SOCS1*, *CBLB*, and *CD5* were identified as key genes that enhance T cell proliferation by upregulating surface CD69 and CD154, revealing therapeutic targets to bolster T cell activation [123]. Through overexpression of a barcoded library containing approximately 12,000 human open reading frames (ORFs), Legut et al. demonstrated that CD4^+^/CD8^+^ T cell proliferation was enhanced by LTBR activation upon TCR stimulation through NF‐κB signaling. The optimized T cell in vivo CRISPR screening platform (OpTICS) was pioneered by Wherry and Shi et al., mechanistically exposing the transcription factor Fli1 as a transcriptional repressor of T_EFF_ responses through dampening of Runx3‐driven activation cascades [240]. Genetic ablation of *Fli1* led to a marked increase in chromatin accessibility at ETS: RUNX DNA‐binding motifs, enabling a 5–30‐fold expansion of the T cell population. Additionally, utilizing genome‐wide CRISPR screening in CD8^+^ T cells both in vitro and in vivo, Zhao et al. discovered that T cell proliferation and antitumor immune responses were significantly enhanced by the depletion of Roquin or overexpression of its target gene *IRF4* [241].

#### 3.2.4 Regulators of T Cell Cytotoxicity

The cytotoxic function of T cells is characterized by the release of granzymes and perforin, as well as interactions between death receptors and their ligands. An in vivo genome‐wide CRISPR screening systematically assessed CD8^+^ T cell functionality in tumor infiltration and degranulation, identifying *Dhx37*, *Lyn*, and *Odc1* as convergent hits. *Dhx37* knockout enhanced CD8^+^ T cell antitumor activity by activating NF‐κB signaling [242]. From the perspective of membrane protein, Chi et al. developed a high‐throughput screen system based on an AAV–SB–CRISPR hybrid vector, enabling efficient gene editing in primary CD8^+^ T cells. Among top hits from three in vivo screens (*Pdia3*, *Mgat5*, *Emp1*), *Pdia3* knockout enhanced metabolic sensitivity in T cells, improving their ex vivo killing efficacy against glioblastoma [134]. As for metabolic pathways, by performing genome‐wide CRISPR screens in murine tumor cells cocultured with cytotoxic T cells (CTLs), Zeng et al. detected that deficiency of two important glycolysis enzymes, *Glut1* (glucose transporter 1) and *Gpi1* (glucose‐6‐phosphate isomerase 1), contributes to enhanced killing of tumor cells by CTLs. Mechanistically, *Glut1* inactivation triggers metabolic reprogramming toward OXPHOS, generating excessive reactive oxygen species (ROS) that potentiate TNF‐α‐mediated tumor cell death in a caspase‐8‐/FADD‐dependent manner [243].

Base editing, a precise CRISPR‐derived gene‐editing technology, enables single‐base substitution without DSBs by fusing Cas9 nickase to cytosine deaminase or adenine deaminase. Leveraging this, a large‐scale base‐editing mutagenesis platform was engineered to interrogate T cell functional genomics, through which a precision‐targeted sgRNA repository (117,000 guides) was constructed across 385 immunologically annotated loci, culminating in the functional mapping of cytotoxicity‐enhancing nodes including *PIK3CD*, *VAV1*, and *DGKZ* via systematic in vitro phenotyping. Subsequent higher‐resolution screen utilizing a base editor with relaxed protospacer‐adjacent motif requirements (NG vs. NGG) revealed the effect of base edits targeting key residues in *PIK3R1* could increase production of TNF, IL‐2, and IFN‐γ [244]. Moreover, critical genes in T cell antitumor regulation had been uncovered via stress‐inducible screening. A genome‐wide CRISPRko screening was conducted upon different stimulations (intense, acute, chronic). As top hits across three screens, *Dap5* ablation enhances translation and tumor killing, while *Icam1* ablation amplifies T cell expansion under both acute and intense stimulation; in contrast, *Ctbp1* inactivation exclusively induces functional persistence during chronic stimulation [245]. Coupled with SLICE technology, A genome‐wide CRISPRko screen subjected to six perturbations (CGS‐21680/tacrolimus/cyclosporine/TGF‐β/T_reg_ coculture/TCR hyperactivation) was conducted in primary human T cells, uncovering RASA2‐deficient clones preserved cytotoxic potency and functional resilience [124].

#### 3.2.5 Regulators of T Cell Exhaustion

Joshi et al. employed in vivo CRISPR screening to assess the role of ∼40 transcription factors (TFs) and epigenetic modulators in T cell fate decisions. It revealed that KLF2 maintains CD8^+^ T effector lineage fidelity by suppressing the exhaustion driver TOX while sustaining core TBET‐driven effector differentiation and a polyfunctional progenitor state, thereby preventing exhaustion [246]. Conversely, Chi et al. identified that multiple transcriptional axes—including IKAROS–TCF–1, ETS1–BATF, and RBPJ–IRF1—drive precursors of exhausted T cells (T_pex_) to exit quiescence and amplify the proliferative capacity of terminally exhausted T cells (T_ex_) [247]. From a clinical perspective, researchers want to enhance the efficacy of ACT and counteract T cell exhaustion in the TME. With CRISPRi and CRISPRa screening on 120 transcriptional and epigenetic regulators, *BATF3* overexpression was found to counter phenotypic and epigenetic signatures of T cell exhaustion upon chronic antigen stimulation [248]. A recent study by Cheng et al. identified KLHL6 as a dual regulator that restricts CD8^+^ T cell exhaustion via two parallel mechanisms. KLHL6 maintains the progenitor exhausted state by degrading the transcription factor TOX, while independently preserving mitochondrial homeostasis by limiting PGAM5‐dependent excessive fission [249].

Altogether, by deconvoluting the genetic determinants of T cell differentiation, activation, cytotoxicity and exhaustion, these screening platforms provide essential mechanistic insights and therapeutic targets for the rational design of next‐generation immunotherapies.

#### 3.2.6 CRISPR Screening Optimizes CAR‐T Therapy

As an innovative antitumor immunotherapeutic approach characterized by precise targeting and manageable toxicity profiles, adoptive cell therapy (ACT) leverages the ex vivo cultivation, expansion, and genetic modification of immune cells for patient reinfusion, thereby demonstrating distinct advantages in amplifying tumor‐suppressive efficacy and overcoming both therapeutic resistance and tumor immune evasion [250]. As the most maturely developed and widely used benchmarking technology in ACT, CAR‐T therapy represents the frontline domain for CRISPR screening implementation in immunotherapy optimization (Table 4).

**TABLE 4:** Research applying CRISPR screening to optimize CAR‐T therapy.

CAR target	Library	Screened cell	Screen model	Discovered target	Published year	References
EGFRvIII	Mouse surface and membrane protein encoding gene library targeting 1658 genes	T cell	In vivo	*PDIA3*	2019	Lupeng Ye [134].
CD19	Whole‐genome CRISPR–Cas9 library	B‐cell lymphoma	In vitro	*FADD*, *RIPK1*	2020	Olli Dufva [251].
CD19	Brie library targets 25 kinases with sustained kinase activity in T cells	T cell	In vitro	*p38 Kinase*	2020	Devikala Gurusamy [252].
BCMA	Whole‐genome CRISPR–Cas9 library	Multiple myeloma (MM) cell	In vitro	*HDAC7*, *Sec61*	2020	Poornima Ramkumar [253].
IL13Rα2	Whole‐genome CRISPR–Cas9 library	CAR‐T cell/glioblastoma stem cells (GSC)	In vitro	*TLE4*, *IKZF2/RELA*, *NPLOC4*	2021	Dongrui Wang [254].
CD19	Whole‐genome CRISPR–Cas9 library	T cell	In vitro	*NAD*	2021	Yuetong Wang [255].
CD19	Whole‐genome CRISPR–Cas9 library	T cell	In vivo	*SOCS1*	2021	Aurélien Sutra Del Galy [256].
CD22; BCMA; HER2	Whole‐genome CRISPR–Cas9 library	T cell	In vitro	*Prodh2*	2022	Lupeng Ye [257].
CD19	Whole‐genome CRISPR–Cas9 library	B‐cell leukemia	In vitro	*NOXA*	2022	Xin Yan [258].
EGFR; IL13Rα2	Whole‐genome CRISPR–Cas9 library	Glioblastoma	In vitro	*IFNγR*	2022	Rebecca C Larson [259].
CD58	Whole‐genome CRISPR–Cas9 library	B‐cell leukemia	In vitro	*CD58*	2022	Xin Yan [260].
B7‐H3	Library targeting 337 epigenetic regulators	T cell	In vivo	*cBAF*	2022	Ao Guo [233].
GD2	Whole‐genome CRISPR–Cas9 library	CAR‐T cell	In vitro	*MED12, CCNC*	2022	Katherine A Freitas [261].
CD19	Whole‐genome CRISPR–Cas9 library	BCP‐ALL	In vitro	*NUDT21*	2022	Matthew T Witkowski [262].
CD19; EphA2	Whole‐genome CRISPR–Cas9 library	T cell	In vitro	*RASA2*	2022	Julia Carnevale [124].
CD19; EGFRvIII	Whole‐genome CRISPR–Cas9 library	B‐ALL	In vitro, in vivo	*IFNγR/JAK/STAT*	2023	Azucena Ramos [263].
HER2	Library targeting 120 TFs and epigenetic modifiers associated with T cell state	T cell	In vitro	*BATF3*	2023	Sean R McCutcheon [248].
HER2	Library targeting 1316 genes that are differentially expressed between in vitro activated vs. naïve CD8 T cells	T cell	In vivo	*ST3GAL1*	2023	Yeonsun Hong [264].
CD22	Cas12a/Cpf1 crRNA library	CAR‐T cell	In vitro	*PRDM1*	2023	Xiaoyun Dai [265].
MSLN	Brunello lentiviral library	Pancreatic carcinoma	In vitro	*GPAA1*, *TFAP4*, *INTS12*	2023	Kimberly R Hagel [266].
CD19	Whole‐genome CRISPR–Cas9 library	CAR‐T cell	In vitro	*Cullin‐5*	2024	Yoshitaka Adachi [267].
CD19	Whole‐genome CRISPR–Cas9 library	T cell	In vivo	*Fas, B2m*	2024	Silvia Menegatti [268].
HER2	Whole‐genome CRISPR–Cas9 library	Cas9 + MC38‐HER2	In vitro	*CBFβ*	2024	Emily J Lelliott [269].
B7‐H3	H1 library	CRISPRi‐U87	In vitro	*ARPC4, PI4KA, ATP6V1A, UBA1, NDUFV1*	2024	Xing Li [270].
CD19	Whole‐genome CRISPR–Cas9 library	Nalm6	In vitro	*ATG3, BECN1, RB1CC1*	2024	Lu Tang [271].
PD‐L1	Brunello CRISPR knockout pooled library containing 77,441 sgRNAs	ATLL	In vitro	*NEDD8, NAE1, UBA3, CUL3*	2024	Masahiro Chiba [272].
CD19	GeCKO v2 library	CAR‐T cell	In vitro	*IL‐4*	2024	Carli M Stewart [273].
CD19	ClinVar library including 8142 ABE sgRNA species to generate variants in 102 genes involved in major T cell functions	CAR‐T cell	In vitro	*PIK3CD*	2025	Zachary H Walsh [274].
CD19	Brunello gRNA library	CAR‐T cell	In vitro	*RHOG*	2025	Paul Datlinger [275].
BCMA	Mario library targeting 135 genes with known or proposed functions in T cells	CAR‐T cell	In vivo	*CDKN1B*	2025	Nelson H Knudsen [276].
CD19	Brunello sgRNA library	Nalm6	In vitro	*NPRL3, NPRL2*	2025	Fuxin Han [277].
PSMA	HKO library	T cell	In vivo	*KMT5A*	2025	Xiaoling Tian [278].

This table summarizes key studies utilizing CRISPR screening to identify genetic modifiers and resistance mechanisms in CAR‐T therapy. Entries are arranged chronologically by publication year.

Contemporary CAR‐T engineering paradigms predominantly employ CRISPR‐based screening centered on T cells and tumor cells as key cellular models. Alternatively, researchers may implement direct genetic perturbations in CAR‐T cells to systematically identify genes critically regulating their functional characteristics, including proliferation, differentiation, and cytotoxic activity, through high‐throughput screening approaches. This dual‐target paradigm expands investigative dimensions while preserving integration efficiency and minimizing off‐target effects in engineered immune cells. However, lentiviral‐mediated delivery of genetic perturbations risks interference with CAR transgene integration. To address this limitation, Chen et al. engineered the CLASH system, which integrates AAV delivery with Cas12a‐mediated genome editing to enable precise, stable knock‐in of large‐scale genetic sequences, effectively circumventing lentiviral‐associated drawbacks [265]. Bock's team developed a high‐content CRISPR screening platform named “CELLFIE” for systematically performing genome‐wide functional genetic screens in human primary CAR‐T cells. This platform integrates the CAR construct and a gRNA library into a single lentiviral vector (CROP‐seq‐CAR), enabling simultaneous CAR transduction and gene editing, and allowing multiplexed phenotypic readouts such as in vitro proliferation, activation, apoptosis, and exhaustion [275].

LOF CRISPR screening studies have systematically identified multiple negative regulators of CAR‐T cell antitumor efficacy, with these genes functionally linked to the modulation of proliferation, effector activity, and cytokine production. A systematic in vitro CRISPR screening targeting 25 kinases in effector cell autonomous functions identified p38 kinase ablation as a potent enhancer of T cell proliferative capacity and antitumor efficacy [252]. A genome‐wide CRISPR screening in effector cells demonstrated that *RASA2* knockout substantially augments CAR‐T cell expansion dynamics, highlighting its therapeutic potential [124]. Adopting a genome‐scale CRISPRko screening in CAR‐T/tumor coculture systems revealed the *MED12/CCNC* complex as a master suppressor, the disruption of which synergistically enhances CAR‐T proliferative kinetics, cytokine release profiles, and cytolytic potential [261]. Functional interrogation of pancreatic cancer cells via genome‐wide Brunello library screening pinpointed *GPAA1*, *TFAP4*, and *INTS12* as key determinants regulating resistance to CAR ‐ T therapy [266]. A genome‐wide CRISPRko screening integrated with RNA‐seq and ATAC‐seq reveals that IL‐4 is a pivotal regulator inducing functional exhaustion in CD8^+^ CAR‐T cells [273]. A genome‐wide CRISPRko screening in human primary CD19 CAR‐T cells identified Cullin 5 (*CUL5*) as a key negative regulator. Its knockout or knockdown significantly enhanced CAR‐T cell proliferation, cytokine secretion, memory phenotype maintenance, and antitumor efficacy [267]. An in vivo CRISPR screening in multiple myeloma xenograft mouse models, employing a custom “Mario” sgRNA library targeting 135 genes with known or proposed functions in T cells for functional screening in BCMA‑targeted CAR‑T cells, identified that loss of *CDKN1B* markedly augments CAR‑T cell proliferation and antitumor activity in vivo without triggering cytokine‑dependent growth or malignant transformation [276]. NPRL2 and NPRL3 were identified in Nalm6 B‐ALL cells as key regulators of CAR‐T resistance, functioning via mTORC1 inhibition to disrupt tumor CAR‐T interactions, impair effector cell activation, and drive immune evasion [277]. Tian et al. performed a high‑content CRISPRko screening focusing on CD8^+^ T cell tumor infiltration and degranulation, identifying *KMT5A* as a negative regulator. They further validated that loss of *KMT5A* enhances CAR‑T cell efficacy against solid tumors [278]. Collectively, these LOF screen discoveries delineate a suppressive regulatory network constraining CAR‐T efficacy, thereby providing a targetable framework for advancing CAR‐T therapeutic development.

GOF screening represents a key strategy for identifying enhancers of CAR‐T cell antitumor efficacy. A genome‐wide GOF screening in T cells systematically identified mitochondria‐localized proline dehydrogenase *PRODH2* as a critical potentiator. The overexpression of *PRODH2* rewires proline metabolism to amplify CAR‐T cell metabolic flux and immunometabolic fitness, ​thereby sustaining durable tumoricidal activity [257]. A GOF CRISPR screening employing a library targeting 120 T cell state‐associated transcription factors and epigenetic regulators revealed that *BATF3* overexpression promotes memory‐like T cell phenotypes while downregulating transcriptional programs linked to cytotoxicity, T_reg_ cell functions, and exhaustion markers [248]. ​Despite the limited quantity of current GOF screening research, their potential to identify targets for enhancing CAR‐T functional efficacy, metabolic regulation, and persistence underscores its scientific importance.

#### 3.2.7 CRISPR Screening Optimizes CAR‐NK Therapy

In the landscape of cancer immunotherapy, NK cells occupy a significant position due to their unique biological attributes. Compared with CAR‐T therapies, CAR‐NK cell‐based approaches exhibit distinct advantages, including broader cellular sourcing, MHC‐unrestricted cytotoxic activity, and reduced risks of cytokine release syndrome and graft‐versus‐host disease [279]. CRISPR screening enables the systematic exploration and optimization of CAR‐NK therapeutic potential. Despite its demonstrated success in CAR‐T functional enhancement, CRISPR screen applications in CAR‐NK research remain disproportionately limited, primarily constrained by NK cell‐specific technical hurdles: low peripheral blood abundance, poor proliferative capacity, and inefficient genetic manipulability. CRISPRa screening focused on cell surface molecules in human leukemia cells identified tumor‐intrinsic *MUC21* overexpression as a potent suppressor of CAR‐NK cell‐mediated tumor clearance [65]. Chen et al. pioneered an AAV‐transposon hybrid system to stably integrate CRISPR libraries into primary NK cell genomes, effectively bypassing the dual technical barriers of NK cell transfection and genome‐editing inefficiency. Multiplexed CRISPR screening coupled with single‐cell transcriptomic profiling revealed *CALHM2* as a previously unrecognized modulator of natural killer cell effector biology, nominating this pathway as a therapeutic target for optimizing CAR‐NK cell potency [89]. Using an in vivo CRISPRa screening followed by ORF‐based validation, Chen's team identified the G protein‑coupled receptor *OR7A10* as a potent enhancer of CAR‑NK therapy. Engineering *OR7A10* into CAR‑NK cells significantly enhanced their efficacy to 100% against multiple solid tumors, representing a promising advance for solid tumor treatment [280]. Rezvani's team performed four distinct CRISPR screens in primary human NK cells, encompassing screens of a transcription factor library, a genome‑wide library, a tumor rechallenge model, and an immunosuppressive stress model. These screens identified several key negative regulators including *MED12*, *ARIH2*, and *CCNC*, whose knockout markedly enhances the antitumor potency of CAR‑NK cells [129]. A genome‐wide CRISPR screening in human NK92 cells identified the fucosyltransferase *FUT8* as an essential gene for NK cell survival under low IL‐15 conditions. Mechanistically, *FUT8* deletion reduced surface expression of the IL‐15 receptor CD122 by 70%, leading to a comprehensive collapse of NK cell viability, proliferation, and cytotoxicity. This suggests *FUT8* as a potential therapeutic target for enhancing NK cell therapies [281]. CRISPR screens in NK‐92 and primary NK cells identified the UBE2F–ARIH2–CRL5 complex as a key regulatory axis controlling IL‐15 receptor (IL‐15R) degradation. Inhibiting this pathway significantly increased IL‐15R surface expression on NK cells, enhanced their antitumor activity, and overcame immunosuppression in the TME, offering a novel strategy to improve CAR‐NK therapy [130]. A genome‐wide CRISPR LOF screening in anaplastic large cell lymphoma revealed that IL‑1 receptor signaling drives the NFKBIZ–IL‐17F–MAPK axis in an autocrine manner to maintain PD‑L1 expression and shape an immunosuppressive TME. Targeted inhibition of this pathway significantly enhances the efficacy of both CAR‑T and CAR‑NK therapies [282].

Compared with CAR‑T therapy, the application of CRISPR screening in the CAR‑NK field remains in its early stages, primarily due to intrinsic technical limitations of NK cells. Their intrinsic antiviral activity leads to substantially lower library delivery efficiency than in T cells [283, 284]. In healthy donors, NK cells typically constitute only about 5–10% of peripheral blood mononuclear cells [285], making it challenging to obtain sufficient starting numbers for subsequent expansion in vitro. Furthermore, effective expansion of NK cells requires a carefully optimized combination of cytokines such as IL‑2, IL‑15, and IL‑18 [286]. The optimization of the cytokine cocktail, concentration ratios, and timing of administration all add complexity, thereby making CRISPR screening in NK cells more challenging. CRISPR screening for CAR‑NK cell optimization remains limited and largely exploratory. Future efforts should prioritize genome editing to enhance persistence, cytotoxicity, and TME infiltration; targeting immune evasion regulators to improve tumor recognition; and reprogramming metabolism to sustain functional longevity. Integrating multidimensional screens with precise gene editing will accelerate the development of potent CAR‑NK therapies.

#### 3.2.8 CRISPR Screening Optimizes Other ACTs

Capitalizing on their superior phagocytic capacity, antigen‐presenting functions, and ability to modulate TME and activate adaptive immunity, macrophages have emerged as a groundbreaking frontier in solid tumor immunotherapy. ​While CRISPR screening remains underutilized in CAR macrophage (CAR‐M) engineering, recent mechanistic studies provide foundational insights: An in vivo CRISPRko screening in TNBC murine models revealed that E3 ubiquitin ligase *Cop1* ablation in tumor cells attenuates macrophage‐associated chemokine secretion, demonstrating suppressed TAM infiltration, enhanced antitumor immunity, and improved response to ICB therapy [68]. Complementary CRISPRko and CRISPRa screening across tumor‐macrophage cocultures by Bassik's team revealed *GPR84* as a macrophage‐specific G protein‐coupled receptor critically mediating enhanced phagocytic clearance of APMAP‐deficient tumors [287], demonstrating CRISPR‐based functional genomics for deconvoluting tumor–immune interactomes in CAR‐M target discovery pipelines. Liu et al. cocultured human ovarian cancer cells carrying a surface protein CRISPR library with CAR‐M cells, revealing that the key autophagy protein ATG9A is central to tumor cell resistance against macrophage killing and elucidating its unique membrane repair mechanism [288]. A Perturb‑map study revealed that ovarian cancer‑secreted IL‑4 directs the formation of an immunosuppressive TME via macrophage control [289]. Although the study did not directly examine CAR‑M, the finding provides a rationale for optimizing CAR‑M therapy: combining it with IL‑4/IL‑4R signaling blockade to neutralize microenvironmental suppression should be considered. A FACS‑based genome‑wide CRISPRko screening in primary mouse macrophages identified *WDFY3* as a regulator of macrophage efferocytosis [128]. This suggests that *WDFY3* or its associated pathway represents a potential target for intrinsic functional enhancement. In designing CAR‑M therapies, overexpressing *WDFY3* may therefore improve therapeutic efficacy.

The application of CRISPR screening in CAR‑M research has been limited, primarily due to the unique biology of macrophages, technical challenges in CRISPR delivery and editing efficiency in primary cells, and the relatively early stage of CAR‑M development compared with CAR‑T technology. Lentiviral vectors are the standard tool for pooled CRISPR screening, yet they exhibit extremely low transduction efficiency in primary macrophages. This resistance is largely attributable to the myeloid‑specific restriction factor *SAMHD1*, which inhibits the viral life cycle by depleting the dNTP pool and degrading viral RNAs [290]. Moreover, macrophages are terminally differentiated cells with limited proliferative capacity in vitro, while CRISPR screening typically requires cell division for stable integration of edits and phenotypic enrichment. The functional complexity of macrophages, including phagocytosis, polarization, and killing poses a challenge for CRISPR screening, as many critical phenotypes lack sensitive, quantitative readouts compatible with high‑content assays, making it difficult to precisely link phenotypes to gene function on a genomic scale. Consequently, editing efficiency is markedly reduced in these nondividing cells. Macrophages exhibit high heterogeneity [291], and their phenotype is highly plastic and sensitive to the microenvironment [292, 293], which can lead to poor reproducibility and high background noise in screening results. To optimize CAR‑M therapy with CRISPR screening, two key directions are essential. First, there is an urgent need to develop more efficient, low toxicity CRISPR delivery systems and screening platforms tailored for macrophages, which is fundamental to overcoming current technical bottlenecks and enabling robust screens. Second, leveraging such platforms, the central research aim should be the systematic discovery of key genetic targets that can enhance CAR‑M antitumor function, persistence, and ability to overcome the immunosuppressive TME.

While CRISPR screening has been preliminarily explored in alternative ACTs including tumor infiltrating lymphocytes (TILs), DC‐based immunotherapy, and γδ T cell‐based approaches, its implementation remains nascent. ​Notably, TIL therapies exhibit unique clinical advantages underscored by their high tumor specificity, favorable safety profiles, scalable expansion potential, and cost‐effective manufacturing, achieving marked efficacy across diverse solid tumors. Both in vitro and in vivo CRISPR screens delineated *SOCS1* as a key regulator of TIL expansion and CD8^+^ T cell intratumoral trafficking, supplying actionable targets to optimize TIL therapeutic potency [294]. Unlike CAR‐T and CAR‐M modalities, DC exert antitumor effects via vaccine‐like strategies by priming cytotoxic T cell responses rather than direct tumoricidal activity. ​Parnas’ team employed a genome‐wide CRISPR screening in murine DC, revealing that dual ablation of CEBPE and *Med12* potentiates DC‐driven T cell proliferation and cytokine secretion [295]. γδ‐T cells participate in tumor immunosurveillance by secreting proapoptotic molecules and inflammatory cytokines or through TCR‐dependent mechanisms. A genome‐wide CRISPRko screening based on the Daudi tumor cell model by Marson's team revealed that AMPK is activated in response to aberrant energy metabolism in tumor cells and promotes γδ‐T cell‐targeted killing activity [296]. While CRISPR screening applications in adoptive cell therapies remain underexplored, the convergence of high‐content screening platforms and mechanistic insights into cellular metabolic regulation positions this technology to deliver innovative molecular targets for therapeutic regimen optimization.

#### 3.2.9 Identification of Novel Immune Checkpoint Targets

Immune checkpoints physiologically maintain immune homeostasis, but their overexpression on tumor cells orchestrates an immunosuppressive TME to evade immune surveillance. Although ICB utilizing monoclonal antibodies have revolutionized oncology across diverse malignancies, their clinical benefit remains constrained by suboptimal response rates and acquired resistance. Therefore, CRISPR screening is urgently needed to discover novel checkpoint targets and resistance mediators to enhance therapeutic efficacy. CRISPR coculture screens with NK cells using MHC‐I^+^ COLO205 colon cancer and MHC‐I^−^ AGS GC cells demonstrated that tumor‐expressed IGSF8 inhibits NK cell degranulation and cytotoxicity via interaction with human *KIR3DL2* or mouse *Klra9* receptors. Currently, anti‐IGSF8 is under investigation as a cancer immunotherapy in a Phase I clinical study in cancer patients (NCT05669430) [297].

In CD8^+^ T cells, using complementary in vivo and in vitro CRISPR–Cas9 genetic screens to target metabolic factors, Chi et al. established voltage‐dependent anion channel 2 (*VDAC2*) as an immune signal‐dependent checkpoint that curtails IFN‐γ‐mediated tumor destruction while concurrently impedes inflammatory reprogramming within the TME to limit antitumor immunity [298]. Beyond directly identifying novel immune checkpoint molecules, CRISPR screening serves as a powerful approach for discovering key upstream regulators that drive checkpoint expression and function. For example, Abdrabou et al., by integrating a high‑throughput microfluidic immunomagnetic cell sorting platform with a genome‑wide CRISPR–Cas9 knockout screen, identified the aryl hydrocarbon receptor (AHR) and IL‑1 receptor‑associated kinase 1 (IRAK1) as core regulators of the immune checkpoint protein VISTA. Inhibiting either AHR or IRAK1 markedly reduced VISTA surface levels on macrophages, reprogramming them from an anti‑inflammatory to a proinflammatory state and thereby enhancing antitumor immunity [299]. A genome‐wide CRISPRko screening based on intracellular staining and flow cytometry revealed the pivotal roles of *IRF1* and *TFAP4* in the transcriptional regulation of the immune checkpoint molecule Galectin‐9, offering a new therapeutic target for leukemia immunotherapy [300].

### 3.3 Developmental and Stem Cell Biology

Developmental and stem cell biology provides insights into tissue formation, disease pathogenesis, and regenerative potential, with precise control of gene expression dictating cell fate decisions. High‐content CRISPR screening has decoded the intricate regulatory networks at unprecedented resolution, systematically investigating gene function even in human models. This section synthesizes recent breakthroughs in developmental and stem cell biology, encompassing cardiovascular development, neurodevelopmental specification, and cell differentiation and regenerative processes.

Ranade et al. leveraged CROP‐seq in trisomy 21 cardiomyocytes derived from human iPSCs to identify HMGN1 as a dosage‐dependent epigenetic regulator driving atrioventricular canal reprogramming toward ventricular fate, solving the challenge of modeling aneuploidy phenotypes in congenital heart disease [301]. Using the same screening method (CROP‐seq) in vascular organoids, researchers from Treutlein's team screened 18 transcription factors and 16 receptors, and revealed *MECOM* as a dual regulator of endothelial and mural cell specification and myofibroblast‐like cells avoidance, which was validated in clonal *MECOM* knockout lines [302].

While challenges existed in extending Perturb‐seq to human PSCs due to transgene silencing, variegated expression, and the complexity of differentiation systems, Sivakumar et al. developed a benchmarked and optimized Perturb‐seq and applied it during human cardiac differentiation to uncover the NKX2‐5–NRG1 axis, a previously uncharacterized regulatory mechanism in human cardiac development, where NKX2‐5 repression of *NRG1* ensures proper ventricular trabeculation [303].

Neurodevelopmental mechanisms are illuminated through CRISPR screening integrated with optical imaging and single‐cell sequencing. To determine pathogenic variants in *SLC6A1* (encoding GABA Transporter 1, GAT‐1) which are associated with developmental delay and avoid radiation harm from traditional [^3^H]‐GABA uptake, McGraw and his colleagues used a high‐throughput fluorescence imaging assay (iGABA‐Snfr) combined with automated microscopy to discover that pathogenic variants in *SLC6A1* strongly reduce GABA uptake, some variants of uncertain significance are also associated with reduced uptake (G111R, S459R, V511M), while 4‐phenylbutyric acid can increase GABA uptake, aiding in the clinical diagnosis and management of NDDs [304]. Human cortical neurogenesis involves complex genetic programs in which radial glia NSCs generate diverse cell types, including excitatory neurons, oligodendrocytes, astrocytes, and interneurons. To systematically examine the regulatory mechanisms governing RG lineage progression, cell fate choice, and subtype specification, a Perturb‐seq screen targeting 44 transcription factors was conducted in human primary cell model, identifying key regulators of neuronal differentiation repression (*ZNF219*), lineage plasticity (*NR2E1* and *ARX*), and subtype specification (*LMO1* and *RIC3*) [305].

High‐content CRISPR screening also plays a pivotal role in uncovering the regulatory mechanisms governing differentiation and regenerative processes. Liang et al. pioneered OSCAR (Organoid‐based Single‐cell CRISPR screening method analyzed with Regulons) to identify *Fos* as a promoter of hepatocyte maturation and *Ubr5* as a maturation delay factor, offering targets for generating functional hepatocytes in vitro [306]. Also using an organoid‐based platform, Lin et al. established a CRISPR screening platform spanning the entire transcription factor repertoire (TFome) in human intestinal organoids, followed by triple fluorescence reporter system coupled with FACS sorting, to systematically identify TFs that govern cell lineage commitment and modulate metabolic activity upon differentiation into enteroendocrine cells (EECs). They identified that *ZNF800* represses enteroendocrine differentiation via direct *PAX4* suppression, the knockout of which leading to increased EEC abundance and decreased goblet and Paneth cell populations [146]. To counteract age‐related NSC activation decline and associated cognitive decline, Ruetz et al. performed a genome‐wide CRISPR screening in primary NSCs from young and old mice (in vitro) and in the aged mouse subventricular zone (in vivo) with Ki67^+^ FACS analysis as high‐content readout, identifying over 300 hits that promote old NSCs to reactivate, among which *Slc2a4* (encoding the glucose transporter GLUT4) knockout emerged as a potent activator that restores neurogenesis by reducing glucose uptake [307]. Complementing studies on physiological NSC aging, high‐content CRISPR screening has further elucidated mechanisms in pathological progeroid syndromes. In Néstor‐Guillermo Progeria Syndrome, an undercharacterized incurable progeroid condition, a genome‐wide CRISPRi screen with multiparametric phenotypic profiling (nuclear shape, micronuclei frequency, nuclear envelope ruptures, and protein mislocalization) in BAF‐mutant patient fibroblasts identified 43 genes (such as NUP160, SEC13, RPL12, RPL37A, LRRK1) whose inhibition rescued nuclear envelope defects, uncovering a mechanism whereby BAF mutation‐induced nuclear permeability changes impair mRNA transport and protein synthesis to drive premature aging [308].

Taken together, these studies have proven high‐content CRISPR screening to be a revolutionary technique for identifying key developmental regulators across various systems. However, a critical gap remains between target identification and functional validation in vivo, with many high‐throughput hits lacking physiological validation. Moreover, approaches detailed in this section predominantly employ single‐cell type perturbations, overlooking multicellular interactions essential for developmental processes, as highlighted by the limited exploration of how key regulators like *HMGN1* affect neighboring cell populations in cardiac development [301]. Future advances require integrating high‐content screening with spatial transcriptomics to capture dynamic cellular interactions in tissue contexts.

### 3.4 Neurobiology

Neurobiology represents a critical frontier where high‐content CRISPR screening demonstrates its transformative potential. This section is structured to progress from NDDs, through neurodegenerative pathologies, and to the neural mechanisms.

#### 3.4.1 Neurodevelopmental Disorders

High‐content CRISPR screening represents a transformative approach in deciphering the genetic complexity of NDDs such as ASD and 22q11.2 deletion syndrome (22q11.2DS) and psychiatric disorders, uncovering convergent molecular pathways and cell‐type‐specific regulatory mechanisms.

Meng et al. conducted pooled CRISPR screening in human forebrain assembloids with complex phenotypic readouts (scRNA‐seq, live‐cell imaging of migration dynamics, and immunohistochemistry) to systematically profile 425 NDD genes, identifying 46 genes involved in human interneuron generation or migration including *CSDE1/SMAD4* for impaired generation and LNPK (an endoplasmic reticulum‐shaping protein) for defective saltatory migration [140]. A key innovation of this study is its establishment of a platform mapping disease genes to specific human neural developmental stages, providing a systematic approach essential for elucidating the biology of complex NDDs and developing effective therapeutics. Similarly, Li et al. developed CRISPR‐human organoids‐scRNA‐seq (CHOOSE) system, which integrates parallel genetic perturbations with single‐cell transcriptomic profiling in mosaic cerebral organoids to identify dorsal intermediate progenitors, ventral progenitors, and upper‐layer excitatory neurons as the most vulnerable cell types in perturbation of 36 high risk ASD genes, while revealing that *ARID1B* perturbation drives ventral progenitor expansion and a fate shift toward oligodendrocyte precursor cells [309]. The first large‐scale multiplex noncoding CRISPRa screening in human iPSC‐derived excitatory neurons targeting cis‐regulatory elements of nine haploinsufficient risk genes, combined with scRNA‐seq for high‐throughput read‐out, identified hit gRNAs that upregulate genes including SCN2A, TBR1, CHD8, and ANK2 via cell‐type‐specific regulatory elements, which are potential treatment targets [310].

Traditional single‐cell CRISPR screening are limited to in vitro applications or specific cell types permissive to lentiviral infection in vivo. To achieve broadly applicable direct in vivo single‐cell screenings, AAV‐Perturb‐seq, an AAV‐based single‐cell or single‐nucleus CRISPR screening method, was developed to dissect 22q11.2DS‐associated genes in the adult mouse prefrontal cortex, revealing that *Dgcr8*, *Dgcr14*, and *Gnb1l* orchestrate RNA processing and synaptic function in mature neurons. This explained approximately 40% of 22q11.2DS transcriptional changes and challenged the notion that these genes only function during development [88]. Another limitation of Perturb‐seq is the loss of spatial context and intercellular interaction information. To address this, Binan et al. developed Perturb‐FISH, an all‐optical high‐content CRISPR screening method that couples in situ gRNA detection with MERFISH to integrate CRISPR perturbation, spatial transcriptomics, and functional phenotypes, enabling the decoding of intracellular, intercellular, and spatial transcriptional circuits, screening 127 ASD risk genes in human iPSC‐derived astrocytes to link perturbations to calcium activity phenotypes and convergent pathways (mTOR signaling, cholesterol metabolism) [176]. Furthermore, Perturb‐seq was optimized by integrating structural topic modeling, an unsupervised machine learning method to reduce the cost of large‐scale Perturb‐seq and enable simultaneous assessment of perturbation effects on cell fate and developmental trajectories using single‐cell readouts. Applying this refined approach to analyze 60 ASD risk genes revealed their function in cell fate specification (*ANKRD11*, *CHD8*, *DEAF1*, *KMT2A*, *KMT2C*, *MBD5*, *MED13L*, *MYT1L*, *NIPBL*, *RFX3*, *TCF4*, *TCF7L2*, and *ZBTB20*) and neuronal differentiation pace (*NF1*, *MED13*, *MBD5*, *TRAF7* and *POGZ*) with ciliogenesis emerging as a key mediating pathway [311]. Complementing these studies, Sun et al. performed the largest‐scale CRISPRi–CROP‐seq screen to date of 87 high‐confidence ASD (hcASD) genes in a human cortical neurogenesis model, identifying 17 genes that alter developmental trajectories (7 and 10 for differentiation deceleration/acceleration, respectively) and demonstrating hcASD convergence on microtubule biology and RNA‐binding proteins [312].

Given the high comorbidity and shared genetic etiology across major psychiatric disorders, the mechanisms underlying pleiotropic effects of risk variants remain to be explored. Utilizing a combination of massively parallel reporter assays and CROP‐seq, Lee et al. discover that pleiotropic genetic variants in psychiatric disorders are regulated by transcription factors with high connectivity in protein–protein interaction networks and exhibit broad chromatin accessibility across excitatory neuronal lineages [313].

Looking ahead, the feasibility of applying high‐throughput CRISPR screening in assembloids has been demonstrated, highlighting the need to explore its application in non‐neural 3D models. Furthermore, while Perturb‐FISH preserves spatial phenotypes within intact tissue architecture, it is limited to targeted gene sets, which necessitates the development of advanced spatial screening techniques for whole‐transcriptome analysis.

#### 3.4.2 Neurodegenerative Diseases

Unlike NDDs that arise from aberrant developmental trajectories, neurodegenerative diseases involve progressive cellular dysfunction and loss in the mature nervous system. This section examines how high‐content CRISPR screening has evolved from static genetic associations toward dynamic functional validation in physiological contexts, covering multiple sclerosis, Alzheimer's disease (AD), and Parkinson's disease (PD).

In multiple sclerosis, macrophages represent the predominant immune cells infiltrating the CNS, yet pooled CRISPR screens have been unable to probe their functions within inflamed tissue environments. de la Rosa et al. overcame this limitation by developing an in vivo CRISPR screening platform combining Perturb‐seq and intravital imaging and screened over 100 cytokine receptors and signaling molecules in Hoxb8FL cells, which revealed that IFNγ, TNF, GM‐CSF, and TGFβ govern macrophage polarization, with TGFβ specifically regulating CNS distribution independent of motility [314].

For AD, Cardona et al. conducted CRISPRi screening of 119 AD GWAS hits in human iPSC‐derived microglia with ROS as a readout, followed by CROP‐seq, identifying *MS4A6A*, *EED*, *INPP5D*, and *RABEP1* as core regulators of microglial inflammation and cell state transitions [315]. Leveraging the 3D iAssembloid coculture system of iPSC‐derived neurons and glia, researchers screened over 2900 kinases and druggable targets as well as neurodegeneration‐related genes from GWASs and revealed that *GSK3b* inhibits NRF2‐mediated protective oxidative stress responses triggered by high neuronal activity; APOE‐ε4‐expressing astrocytes induce neuronal hyperactivity compared with APOE‐ε3 astrocytes, unravelling the mechanisms of neuron–glia interaction [316].

Transplantation was demonstrated as a potential therapy for PD, but the extensive cell death following transplantation emerged as a critical challenge. Kim et al. applied an in vivo CRISPR screening targeting cell death pathway genes in dopamine neurons, followed by FACS, to identify the TNF–NF‐κB–p53 axis as the key limiter of neuronal survival posttransplantation. Targeting this axis through TNF‐α inhibition (adalimumab) and CD49e‐low sorting rescued neuronal survival, establishing a direct therapeutic strategy for PD [317].

Collectively, these studies recapitulated the multicellular microenvironment and disease‐relevant stressors utilizing high‐content CRISPR screening. However, in vivo screening remains limited to focused gene libraries rather than genome‐wide scale [314], while iPSC‐based platforms, despite capturing neuron–glia interactions, predominantly model acute pathological responses rather than chronic neurodegenerative progression [315].

#### 3.4.3 Neural Mechanisms

Different from the disease‐focused screenings, this subsection shifts toward uncovering basic neural mechanisms governing brain development, cell identity specification, and intercellular communication, which represent fundamental questions in neuroscience.

Zheng et al. established a high‐throughput in vivo Perturb‐seq platform using AAV‐SCH9 and a transposon system to map cell‐type‐specific transcriptional networks during cortical development, revealing *Foxg1's* critical role in maintaining Layer 6 corticothalamic neuron identity by repressing alternative lineage programs [87]. Reisman et al. integrated CRISPRa with multiomics to identify *INSM1* as a master regulator for astrocyte‐to‐neuron reprogramming, with *IKZF1* acting as a synergistic cofactor through chromatin remodeling at synaptic gene promoters [318]. Pushing the field further, Shen et al. introduced spatial Perturb‐Seq, which combines CRISPR perturbations with spatial transcriptomics to dissect both cell‐autonomous transcriptional changes and noncell‐autonomous microenvironmental effects within intact tissue architecture [319]. To clarify the molecular basis of presynaptic homeostatic potentiation (PHP), Martinez et al. conducted a comprehensive in vivo CRISPR/Cas9‐based screen of the Drosophila glutamate receptome using the larval neuromuscular junction model, revealing that the kainate receptor subunit Ekar acts as a selective, critical regulator of chronic PHP.

Collectively, these insights into neural mechanisms lay the groundwork for dissecting neurological disorders. Currently, these approaches remain unable to scale up sufficiently to capture the full complexity of neural circuits and their long‐term functional consequences.

### 3.5 Infectious Diseases

In viral infection studies, the application of high‐content CRISPR screening has evolved from single‐cell transcriptomic profiling to image‐based analysis. Sunshine et al. utilized Perturb‐seq, combining CRISPR interference with single‐cell transcriptomics to interrogate 183 SARS‐CoV‐2 host factors in human lung epithelial Calu‐3 cells and identified *NFKBIA*, *EIF4E2*, and *EIF4H* as strong host dependency factors, with *EIF4H* knockout reducing infection by 33.2% compared with nontargeting controls [320]. In another study aimed at decoding factors involved in the IFN response under SARS‐CoV‐2 infection, researchers employed a genome‐wide knockout screen in Huh‐7.5 cells and used nucleoprotein immunofluorescence staining and high‐content microscopy as readout. Among the top hits, *PLSCR1* was identified as one of the most potent antiviral genes and served as an intrinsic entry barrier against SARS‐CoV‐2 entry by blocking the endocytic pathway, a mechanism that Omicron variants have evolved to evade [321]. Further advancing the field, CarlAson et al. employed a similar readout approach as described above, but further incorporated machine learning classifiers to capture multidimensional and subtle phenotypes, identifying host factors affecting viral entry, replication, and virus‐induced cellular morphological changes [322].

For parasitic infections, Marzook et al. screened over 18,000 human protein‐coding genes in HCT‐8 cells and extracted four infection hallmarks (parasite growth, parasite development, actin pedestal recruitment, and host cell viability) with the aid of high‐content confocal microscopy, revealing that Cryptosporidium survival hinges on host squalene reserves. Crucially, they demonstrated that the squalene synthase inhibitor lapaquistat effectively restricts Cryptosporidium infection in mice, providing a promising therapeutic strategy for a disease with limited treatment options [323].

Overall, high‐content CRISPR screening has proven to be a powerful tool for uncovering host–pathogen interactions. However, this field remains significantly underdeveloped compared with aforementioned applications in cancer biology, immunology, and developmental biology.

### 3.6 Evaluation of CRISPR Screening Targets in Clinical Trials

Although CRISPR screening has identified a multitude of potential targets in the laboratory, not all “essential genes” uncovered translate into viable drug targets due to complex clinical challenges such as safety profiles, delivery efficiency, and pharmacokinetic properties. This section aims to bridge this gap by systematically reviewing cases where CRISPR screen‐identified targets have progressed to clinical evaluation. These translational efforts can be categorized into three pivotal areas: identifying druggable targets, repurposing of existing drugs, and uncovering biomarkers that are now being assessed in clinical trials.

#### 3.6.1 CRISPR Screening for Direct Drug Targets

In 2017, Manguso et al. demonstrated through an in vivo CRISPR screening that loss of *PTPN2* enhances tumor sensitivity to immunotherapy [212]. Building on this finding, the first active‐site inhibitor of *PTPN2/PTPN1*, ABBV‐CLS‐484 (AC484), entered a Phase I clinical trial in 2021 (NCT04777994), where it was shown to enhance NK and CD8^+^ T cell function, reduce T cell exhaustion, and elicit potent antitumor immunity in PD‐1‐resistant models [211]. Subsequently, next‐generation inhibitors K‑38 and WS35 have been developed with improved oral bioavailability and have demonstrated promising efficacy in melanoma models [324, 325].

Beyond directly targeting immunoregulatory pathways, CRISPR screening has also achieved breakthrough progress in the field of DDR. In 2019, Wellcome Sanger Institute performed systematic genome‐wide CRISPRko screens across 324 cancer cell lines and identified *WRN* as a synthetic lethal target in multiple MSI‐H cancer cells, where *WRN* knockout induced accumulation of DNA DSBs, chromosome fragmentation, and cell death selectively, while no significant effect on microsatellite‐stable cells [190]. The large‐scale CRISPR and RNAi screen by Chan et al. elucidated its mechanism that MSI‐H cancer cells critically depend on *WRN* helicase activity for genome maintenance, due to expanded TA‐dinucleotide repeats and replication stress led by mismatch repair deficiency [326]. Beyond conventional genome‐wide knockout screens, advanced genome editing tools have further refined the understanding of *WRN* as a drug target. Picco et al. provide a detailed account of the biochemical, cellular and in vivo activities of novel selective *WRN* helicase inhibitors, offering multiple lines of evidence supporting the clinical development of *WRN* inhibitors [327].

Based on these discoveries, WRN inhibitors have entered clinical development via distinct mechanisms, notably Novartis's allosteric inhibitor HRO761 (NCT05838768), which demonstrated acceptable safety and durable efficacy in advanced MSI‐H/dMMR cancers [328]., which demonstrated acceptable safety and durable efficacy in advanced MSI‐H/dMMR cancers, and Vividion Therapeutics’ covalent inhibitor (NCT06004245) targeting the C727 cysteine residue [329, 330].

Gallo et al. performed genome‐scale CRISPR screens in *CCNE1*‐high clones and first identified *PKMYT1* maintain G2/M checkpoint stability to counteract the replication stress induced by *CCNE1* amplification, thereby preventing DNA damage accumulation and mitotic catastrophe [331]. *PKMYT1* loss exerts lethal effects selectively in tumor with CCNE1 amplification, which is prevalent in multiple tumor types, particularly in high‐grade serous ovarian cancer, uterine tumor, and gastro‐esophageal cancer. Using a structure‐guided medicinal chemistry approach, the research team developed RP‐6306, an orally bioavailable and selective inhibitor that shows single‐agent activity and durable tumor regressions when combined with gemcitabine in models of *CCNE* amplification. With its entry into Phase I clinical trials (NCT05147272, NCT05147350), RP‐6306 has become the first *PKMYT1* inhibitor to reach clinical evaluation.

Genome‐wide CRISPR screening conducted by Patterson‐Fortin et al. demonstrate that simultaneous disruption of two DNA repair pathways, NHEJ and MMEJ, via combined treatment with DNA‐PK inhibitor peposertib plus a POLθ inhibitor novobiocin exhibited synergistic synthetic lethality specifically in TP53‐mutant tumors resulting from accumulation of toxic levels of DNA DSB end resection [332]. Although this combination strategy remains in the preclinical stage, with recent entry of POLθ inhibitors such as ART4215, GSK4524101, and SIM0508 into clinical trials positions, dual inhibition of NHEJ and MMEJ is expected as a promising candidate for future clinical evaluation in *TP53*‐mutant malignancies.

Notably, the druggable targets uncovered by CRISPR screening extend beyond tumor cell‐intrinsic vulnerabilities, supplying a rich repertoire of actionable targets to optimize the TIL therapeutic potency. Both in vitro and in vivo CRISPR screens under TIL expansion conditions delineated *SOCS1* as the top hit driving TIL expansion and CD8^+^ T cell intratumoral trafficking. A comprehensive CRISPR tiling screen of the *SOCS1* coding region was performed to precisely target the SH2 domain, identifying an sgRNA with minimal off‐target cuts and optimal editing efficiency for manufacturing KSQ‐001, a CRISPR–Cas9 engineered TIL therapy with *SOCS1* inactivation [294]. KSQ‐001 entered first‐in‐human Phase I/II clinical trials in 2024 (NCT06598371), evaluating its safety and efficacy in patients with advanced melanoma, NSCLC, and HNSCC. Concurrently, KSQ has developed KSQ‐004EX, a next‐generation eTIL therapy with dual *SOCS1*/Regnase‐1 editing; preclinical data demonstrate > 94% editing efficiency, enhanced cytotoxicity, increased IFN‐γ production, and improved in vivo function and persistence in NSG mouse models [333].

#### 3.6.2 CRISPR Screening for Drug Repurposing Targets

The advent of CRISPR screening has substantially expanded the horizon of drug repurposing, beyond the discovery of novel targets, it enables systematic dissection of new indications for approved drugs. Liu et al. integrated single‐cell multiomics with genome‐wide CRISPR screening and discovered that genes upregulated in interneuron lineage progenitors constitute major dependencies in *H3G34*‐mutant diffuse hemispheric glioma (DHG‐H3G34), with *CDK6* emerging as a targetable vulnerability. Guided by these findings, the research team assessed a range of *CDK4/6*‐inhibitors approved for breast cancer treatment (ribociclib, palbociclib, and abemaciclib) and *CDK6*‐specific degrader BSJ‐03‐123 in multiple patient‐derived HGG cell lines, which promoted a shift toward more mature interneuron‐like states, reduced tumor growth, and prolonged xenograft survival. Highlighting this translational potential, compassionate ribociclib treatment achieved 17 months of progression‐free survival in a twice‐relapsed pediatric DHG‐H3G34 patient, successfully repurposing this breast cancer drug for refractory brain tumors [334]. Leveraging established safety profiles, drug repurposing significantly accelerates bench‐to‐bedside timelines. As CRISPR screening technologies continue to advance and more compounds are incorporated into screening libraries, this strategy holds promise for delivering rapid and cost‐effective therapeutic solutions for diseases currently lacking effective treatments, ultimately realizing the clinical value of “repurposing with precision.”

#### 3.6.3 CRISPR Screening for Biomarker Discovery and Patient Stratification

CRISPR screening via performing genome‐wide functional screens under specific drug pressures, systematically identifies genes directly associated with drug response, uncovering functional biomarkers that truly drive therapeutic outcomes. These biomarkers not only offer superior predictive accuracy but can also be directly translated into clinically actionable stratification strategies, guiding precision medicine decisions.

A large‐scale CRISPR–Cas9ko screening of colorectal cancer cell lines systematically analyzed 17 colorectal cancer cell lines and 513 other cancer cell lines, identifying 1828 colorectal cancer‐specific dependency genes. A *CFG22* scoring model was then constructed. Further drug sensitivity analysis revealed that patients with high *CFG22* scores are more sensitive to the repurposed lipid‐lowering drug clofibrate, providing a clear direction for personalized treatment [335]. Yap et al. utilized its genome‐wide CRISPR screening platform to uncover and confirm synthetic lethal sensitizing gene alterations for the ATR inhibitor camonsertib (RP‐3500). The Phase I TRESR study (NCT04497116) enrolled 120 patients with advanced solid tumors harboring DDR gene alterations (LOF of *ATM*, *ATRIP*, *BRCA1*, *BRCA2*, *CDK12*, *CHTF8*, *FZR1*, *MRE11*, *NBN*, *PALB2*, *RAD17*, *RAD50*, *RAD51B/C/D*, *REV3L*, *RNASEH2A*, *RNASEH2B*, or *SETD2*). Across all tumor and molecular subtypes, 12% (13 out of 113) had a protocol‐defined tumor response and the clinical benefit rate was 42% (47 out of 113). Enhanced efficacy was particularly observed in patients harboring biallelic alterations [336].

CRISPR screening is reshaping the paradigm of drug target discovery. From its early applications in defining gene essentialness in cell lines to directly guiding target development and biomarker stratification in clinical trials, CRISPR screening has bridged the gap between fundamental research and clinical translation. This translational pathway has been concretely demonstrated in multiple cases that have advanced to clinical development, with the relevant trial information summarized in Table 5. These cases collectively delineate a clear translational roadmap that unbiased genome‐wide screening establishes causal genotype–phenotype linkages, high‐content functional readouts and structural biology guide drug development, and clinical trials ultimately validate their therapeutic value. With increasing screening throughput, richer functional readouts, and optimized translational pathways, we anticipate that the coming decade will witness a growing number of CRISPR‐identified targets advancing into clinical practice, truly realizing the vision of “from gene to medicine” with precision. However, despite this promising translational trajectory, several profound technical and systemic hurdles must still be overcome to fully unlock the clinical and scientific potential of these screening platforms.

**TABLE 5:** Targets and drugs discovered through CRISPR screening and advanced into clinical development.

Drug	Introduction	Target	ClinicalTrials.gov ID	Phase	CRISPR screening findings
ABBV‐CLS‐484	ABBV‐CLS‐484 is an oral *PTPN2/PTPN1* inhibitor that enhances antitumor immunity by boosting JAK–STAT signaling and reducing T cell dysfunction.	*PTPN2/PTPN1*	NCT04777994	Phase 1 study	In vivo CRISPR screening identified that deletion of *PTPN2* in tumor cells augments interferon‐gamma signaling to potentiate responses to immune checkpoint blockade [212].
HRO761	HRO761 is an oral drug that acts on WRN, which may contribute to cancer growth. By acting on *WRN*, HRO761 may be able to stop the growth of the cancer.	*WRN*	NCT05838768	Phase 1 study	A genome‐wide CRISPR screening across 324 cancer cell lines established a data‐driven target prioritization framework and validated *WRN* helicase as a synthetic lethal target in MSI tumors [190].
VVD‐133214	VVD‐133214 is an oral drug that acts on *WRN*, which may promote the growth of cancers that are MSI and/or dMMR. By acting on *WRN*, VVD‐133214 may be able to block the growth of these types of cancer.	*WRN*	NCT06004245	Phase 1 study	A genome‐wide CRISPR screening across 324 cancer cell lines established a data‐driven target prioritization framework and validated *WRN* helicase as a synthetic lethal target in MSI tumors [190].
GSK4418959	GSK4418959 is a noncovalent, reversible, selective, orally active *WRN* helicase inhibitor that suppresses the ATPase and DNA unwinding activities of *WRN* helicase through an ATP‐competitive mechanism.	*WRN*	NCT06710847	Phase 1/2 study	A genome‐wide CRISPR screening across 324 cancer cell lines established a data‐driven target prioritization framework and validated *WRN* helicase as a synthetic lethal target in MSI tumors [190].
RP‐6306/lunresertib	RP‐6306 is an effective, selective, orally active *PKMYT1* inhibitor.	*PKMYT1*	NCT04855656	Phase 1/1b study	This CRISPR screening revealed that inhibiting the *PKMYT1* kinase in cells with *CCNE1* gene amplification results in a synthetic lethal effect [331].
RP‐6306/lunresertib	RP‐6306 is an effective, selective, orally active *PKMYT1* inhibitor.	*PKMYT1*	NCT05605509	Phase 2 study	This CRISPR screening revealed that inhibiting the *PKMYT1* kinase in cells with *CCNE1* gene amplification results in a synthetic lethal effect [331].
RP‐6306/lunresertib	RP‐6306 is an effective, selective, orally active *PKMYT1* inhibitor.	*PKMYT1*	NCT05147272	Phase 1 study	This CRISPR screening revealed that inhibiting the *PKMYT1* kinase in cells with *CCNE1* gene amplification results in a synthetic lethal effect [331].
RP‐6306/lunresertib	RP‐6306 is an effective, selective, orally active *PKMYT1* inhibitor.	*PKMYT1*	NCT05147350	Phase 1 study	This CRISPR screening revealed that inhibiting the *PKMYT1* kinase in cells with *CCNE1* gene amplification results in a synthetic lethal effect [331].
RP‐3500/camonsertib	RP‐3500 is an orally active, highly selective ATR kinase inhibitor intended to treat advanced solid tumors harboring specific DDR gene LOF alterations.	*ATR*	NCT04497116	Phase 1/2a study	Internal and external CRISPR screening datasets have collectively identified a set of DDR pathway genes whose LOF alterations predict potential tumor sensitivity to the ATR inhibitor camonsertib [336, 337, 338].
ART4215	ART4215 is a potent and selective inhibitor of DNA polymerase (pol) theta.	*Polθ*	NCT04991480	Phase 1/2a study	A CRISPR screening revealed that the combined inhibition of the NHEJ and MMEJ pathways induces a synthetic lethal effect in *TP53*‐mutated tumors [332].
ART6043	ART6043 is an oral anticancer agent in combination with a poly (adenosine diphosphate ribose) polymerase (PARP) inhibitor (PARPi) in patients with cancers that harbor defects in DNA repair.	*Polθ*	NCT05898399	Phase 1/2a study	A CRISPR screening revealed that the combined inhibition of the NHEJ and MMEJ pathways induces a synthetic lethal effect in *TP53*‐mutated tumors [332].
GSK4524101	GSK4524101 is an oral DNA polymerase theta (*POLθ*) inhibitor.	*Polθ*	NCT06077877	Phase 1/2 study	A CRISPR screening revealed that the combined inhibition of the NHEJ and MMEJ pathways induces a synthetic lethal effect in *TP53*‐mutated tumors [332].
SIM0508	SIM0508 is a potent and selective small‐molecule inhibitor targeting *Polθ*.	*Polθ*	NCT06686745	Phase 1 study	A CRISPR screening revealed that the combined inhibition of the NHEJ and MMEJ pathways induces a synthetic lethal effect in *TP53*‐mutated tumors [332].
KSQ‐004EX	KSQ‐004EX is an engineered tumor‐infiltrating lymphocyte therapy that inactivates both *SOCS1* and Regnase‐1 via CRISPR/Cas9 for the treatment of advanced solid tumors.	*SOCS1*	NCT06598371	Phase 1/2 study	A CRISPR screening discovered that the *SOCS1* gene is a key checkpoint that restrains T cell antitumor function, and that its inactivation significantly enhances the in vitro and in vivo activity of tumor‐infiltrating lymphocytes [294].
KSQ‐001EX	KSQ‐001EX is an engineered tumor‐infiltrating lymphocyte therapy that inactivates the *SOCS1* gene via CRISPR/Cas9 for the treatment of advanced solid tumors.	*SOCS1*	NCT06237881	Phase 1/2 study	A CRISPR screening discovered that the *SOCS1* gene is a key checkpoint that restrains T cell antitumor function, and that its inactivation significantly enhances the in vitro and in vivo activity of tumor‐infiltrating lymphocytes [294].

## 4 Challenges and Future Directions

### 4.1 Scaling, Cost, and Standardization

Large‐scale CRISPR screens, particularly genome‐wide​ studies, are highly resource‐intensive and costly. Genome‐wide CRISPR screens necessitate the transduction of hundreds of millions to billions of cells to ensure adequate gRNA representation and maintain single‐guide occupancy (typically via low MOI) [70, 339]. This massive cellular demand renders such screens prohibitively labor‐intensive and cost‐prohibitive, often rendering them technically infeasible​ for rare primary cell populations or clinically relevant patient‐derived samples where cell numbers are limiting. Beyond the challenge of cell procurement, the financial burden of performing high‐content readouts is also substantial. While multimodal high‐content readouts provide unparalleled phenotypic depth, their exorbitant operational costs represent the absolute bottleneck limiting the scalability of high‐content CRISPR screening. Whether utilizing flow cytometry‐based high‐content screening, image‐based high‐content CRISPR screening, single‐cell sequencing‐coupled CRISPR screening, or high‐content readout beyond transcriptomics, the application of these modalities is restricted by both high data‐acquisition costs and technical incompatibilities with certain cell types. For instance, fragile primary neurons or suspension immune cells often cannot tolerate the processing required for optical imaging screens, while complex organoids can suffer lethal cellular stress during the enzymatic dissociation needed for single‐cell sequencing [159, 340]. Addressing these limitations, the Steinmetz's team developed targeted Perturb‐seq (TAP‐seq). Rather than capturing the entire transcriptome, TAP‐seq focuses on the quantitative analysis of hundreds of transcripts of interest, substantially reducing the cost of single‐cell perturbation screens [341].

CRISPR screening inherently relies on deploying multiple gRNAs per gene to elicit consistent and concordant perturbation signals. However, profound variability in editing efficiencies across distinct genomic loci and individual gRNA sequences inevitably introduces technical noise, giving rise to both false‐negative and false‐positive results [23]. To address this bottleneck, a diverse array of statistical and computational models has been developed to accurately predict gRNA performance and systematically account for this editing heterogeneity [342, 343, 344, 345]. Concurrently, off‐target activity remains a persistent concern in CRISPR screening, as gRNAs can mistakenly target noncanonical genomic sites and lead to unexpected phenotypes. This issue is particularly prominent for noncleaving modalities like CRISPRi and CRISPRa, where off‐target effects can be more severe than in traditional CRISPRko [346]. To address this, AI‐assisted gRNA design has substantially helped mitigate these nonspecific interactions [347, 348]. Additionally, various experimental confounding factors can interfere with the interpretation of CRISPR screening results. These include nonuniform MOI [339], as well as clonal selection and growth biases during long‐term culture [349]. Consequently, the observed phenotypes may not be directly caused by the target gene perturbation, but may instead represent experimental artifacts.

Although numerous guidelines have been published, CRISPR screening still lacks unified standards [70, 339, 350, 351, 352]. This challenge persists throughout the entire workflow, manifesting in the absence of universal criteria for gRNA and library design, reliance on empirical experience for experimental execution and model selection, and a diverse array of data processing and analytical tools without established consensus. Reproducibility and data sharing are key components of standardization of CRISPR screening. Several public CRISPR screening databases and portals are highlighted as useful resources for reusing screening results and discovering targets, including The Cancer Dependency Map [353], the database of Pooled In vitro CRISPR Knockout Library Essentiality Screens [354], integrated Database of CRISPR Screens [355], Project Score database [356], The Biological General Repository for Interaction Datasets (BioGRID ORCS) [357], GenomeCRISPR [358], and CRISP‐view [359]. Despite the ongoing challenges in standardizing CRISPR screening, emerging public databases and resources offer practical tools to enhance reproducibility, facilitate data sharing, and drive consensus toward unified protocols for robust discovery.

### 4.2 Integration of Spatial and Temporal Dimensions

Single‐cell sequencing‐coupled CRISPR screening requires tissue dissociation, which eradicates the native spatial context critical for interpreting perturbation phenotypes in disease [12, 15]. In contrast, spatial transcriptomics preserves positional information but cannot define genetic causality. Bridging these complementary yet disjointed modalities remains a major technical and analytical challenge. Perturb‐FISH combines imaging‐based spatial transcriptomics with optical in situ detection of CRISPR perturbations, enabling single‐cell resolution studies of genetic and molecular associations within spatial and functional biology [176]. SPAC‐seq simultaneously captures CRISPR perturbation barcodes and whole‐transcriptome profiles while preserving native tissue architecture [360]. CLIM–TIME integrates in vivo CRISPR screening with LCM, enabling spatially resolved analysis of TME [178]. Moreover, the inherently destructive nature of scRNA‐seq precludes longitudinal tracking of the same cell, and this absence of a temporal dimension significantly constrains the study of cellular dynamics. Live‐seq employs fluidic force microscopy to maintain cell viability during RNA extraction, enabling longitudinal single‐cell transcriptomic profiling as well as downstream molecular and functional analyses of the same cell across multiple time points [361]. Integrating Live‐seq with CRISPR screening enables real‐time tracking of dynamic transcriptional changes following gene perturbations within the same cell, offering a powerful approach particularly suited for time‐sensitive investigations.

Collectively, the integration of spatial and temporal resolution into CRISPR screening addresses a critical bottleneck: the lack of applicable screening models for many complex diseases. Conditions such as solid tumors and fibrotic disorders have long been unsuitable for conventional high‐throughput screens, as their pathogenesis strictly depends on intact microenvironments and dynamic state transitions that are lost in dissociated cultures. By preserving tissue architecture and enabling longitudinal tracking, spatiotemporally resolved CRISPR approaches allow genetic perturbations to be evaluated in more physiologically relevant settings.

### 4.3 Clinical Translation Potential

CRISPR screening technologies mature as a platform for target discovery identifying numerous candidate genes whose modulation may hold therapeutic potential for diseases such as cancer, cardiovascular disorders, and neurodegeneration. However, the journey from genetic hits discovered by screening to clinically approved drugs is a long and uncertain translational path.

Transitioning CRISPR screening from in vitro cell cultures to in vivo and organoid preclinical models is an essential step for validating target clinical potential. However, the safety of delivery systems remains a major hurdle. Regarding clinical translational potential, AAV and exosomes offer the greatest safety advantages due to minimal immunogenicity and favorable biocompatibility. While LNPs and RNPs avoid genomic integration risks and exhibit low off‐target effects, they face challenges including material‐induced inflammatory responses and cytotoxicity from physical delivery, respectively. In contrast, LVs remain disadvantaged in clinical safety assessments owing to risks of insertional mutagenesis and immunogenicity [362]. Beyond engineering safer and more efficient vectors, refining screening strategies is equally critical. To circumvent in vivo vector toxicity associated with large library delivery, the prevailing logic should shift from unfocused, genome‐wide in vivo screens toward a sequential approach: first narrowing candidates via in vitro screening to construct a focused library, followed by low‐dose in vivo validation. This strategy optimally balances safety profiles with screening depth [70, 363].

CRISPR screens must capture patient heterogeneity to yield clinically actionable targets. To address patient heterogeneity in clinical translation, future screening strategies should transition from reliance on a limited number of immortalized cell lines or model mice to performing CRISPR screening in patient‐derived organoids that preserve the native heterogeneity of individual patients [364, 365]. Heterogeneity extends beyond interpatient variation to encompass intrinsic differences among distinct cell types within the complex TME. The future of CRISPR screening lies in spatially resolved, in vivo multicellular perturbations, shifting the focus from identifying isolated tumor targets to comprehensively dissecting the complex ecosystem of the TME [360].

## 5 Conclusions and Perspectives

High‐content CRISPR screening has definitively transitioned functional genomics from static, unidimensional observations to the systematic dissection of complex, dynamic biological mechanisms. By integrating programmable CRISPR perturbations with cutting‐edge multimodal profiling technologies, this transformative approach allows researchers to resolve intricate cellular heterogeneity and dynamic intercellular crosstalk at single‐cell resolution. It has revolutionized our understanding of sophisticated biological systems, proving particularly invaluable in dissecting the TME, mapping neural circuits, and elucidating immune cell interactions.

Despite this immense progress, the field must navigate several critical bottlenecks to fully realize its potential. The success of both screening precision and subsequent clinical translation relies heavily on the continued development of efficient and safe in vivo delivery methods. Currently, viral vectors remain indispensable due to their high transduction efficiency, yet they are constrained by packaging limits, immunogenicity, and the risk of insertional mutagenesis. Conversely, nonviral vectors, such as LNPs, offer improved safety profiles and reduced genomic integration risks, but their inability to sustain long‐term expression limits their utility in pooled screens focused on proliferation.

With the continuous optimization of delivery platforms, the integration of artificial intelligence for massive data deconvolution, and the expansion of in vivo screening models, high‐content CRISPR screening is poised to remain a cornerstone of biomedical discovery for the next decade, truly realizing the clinical vision of moving “from gene to medicine” with unprecedented precision.

## Author Contributions

Y.Z., Y.Z., X.T., X.D., and Y.Z. wrote the review. J.L. and Y.W. conceived and designed this review. J.L. provided direction and guidance throughout the manuscript preparation. All authors have read and approved the final manuscript.

## Funding

This work was supported by grants from the Shanghai Natural Science Foundation (25ZR1402572) and the National Natural Science Foundation of China (32500805).

## Ethics Statement

The authors have nothing to report.

## Conflicts of Interest

The authors declare no conflicts of interest.

## Data Availability

No datasets were generated or analyzed during the current study.
